# Multiplexed mapping of the interactome of GPCRs with receptor activity–modifying proteins

**DOI:** 10.1126/sciadv.ado9959

**Published:** 2024-07-31

**Authors:** Ilana B. Kotliar, Annika Bendes, Leo Dahl, Yuanhuang Chen, Marcus Saarinen, Emilie Ceraudo, Tea Dodig-Crnković, Mathias Uhlén, Per Svenningsson, Jochen M. Schwenk, Thomas P. Sakmar

**Affiliations:** ^1^ Laboratory of Chemical Biology and Signal Transduction, The Rockefeller University, New York, NY, USA.; ^2^ Tri-Institutional PhD Program in Chemical Biology, New York, NY, USA.; ^3^ Science for Life Laboratory, School of Engineering Sciences in Chemistry, Biotechnology and Health, KTH Royal Institute of Technology, Solna, Sweden.; ^4^ Department of Clinical Neuroscience, Karolinska Institutet, Stockholm, Sweden.; ^5^ Basal and Clinical Neuroscience, King’s College London, London, UK.; ^6^ Department of Neurobiology, Care Sciences and Society, Section for Neurogeriatrics, Karolinska Institutet, Solna, Sweden.

## Abstract

Receptor activity–modifying proteins (RAMPs) form complexes with G protein–coupled receptors (GPCRs) and may regulate their cellular trafficking and pharmacology. RAMP interactions have been identified for about 50 GPCRs, but only a few GPCR-RAMP complexes have been studied in detail. To elucidate a comprehensive GPCR-RAMP interactome, we created a library of 215 dual epitope-tagged (DuET) GPCRs representing all GPCR subfamilies and coexpressed each GPCR with each of the three RAMPs. Screening the GPCR-RAMP pairs with customized multiplexed suspension bead array (SBA) immunoassays, we identified 122 GPCRs that showed strong evidence for interaction with at least one RAMP. We screened for interactions in three cell lines and found 23 endogenously expressed GPCRs that formed complexes with RAMPs. Mapping the GPCR-RAMP interactome expands the current system-wide functional characterization of RAMP-interacting GPCRs to inform the design of selective therapeutics targeting GPCR-RAMP complexes.

## Teaser

A multiplexed screen reveals complexity in the interactome between GPCRs and receptor activity–modifying proteins (RAMPs).

## INTRODUCTION

Protein-protein interactions regulate signal transduction by heterotrimeric guanine nucleotide–binding regulatory proteins (G proteins). G protein–coupled receptors (GPCRs), which comprise a superfamily of approximately 720 distinct receptors, activate G proteins in response to ligand binding. Receptor activity–modifying proteins (RAMPs) have been shown to regulate GPCR trafficking and ligand specificity for several receptors, including the calcitonin receptor–like receptor (CALCRL) (*1*, *2*). Several single-particle cryo–electron microscopy structures of GPCR-RAMP complexes show them as bimolecular pairs (*1*, *3*–*6*). Bioinformatic studies show that RAMPs globally coevolved with GPCRs (*7*), and concordance between GPCR and RAMP transcript levels has been observed (*8*). Consequently, elucidation of the GPCR-RAMP protein interactome has important implications in understanding the cell biology and pharmacology of GPCR signaling and for drug discovery programs that target GPCRs.

We have previously reported an affinity proteomic study of GPCR-RAMP complexes in the secretin-like subfamily of GPCRs. We determined the interactome of 23 GPCRs with three RAMPs and showed that most secretin family GPCRs interact with at least one RAMP (*9*). In addition, cell-based bioluminescence energy transfer–based assay screens demonstrated that several chemokine GPCRs interact with at least one RAMP (*10*). These studies suggest that GPCR-RAMP interactions might be widespread, but a systematic investigation of the expanded GPCR-RAMP interactome has yet to be reported.

We report the GPCR-RAMP interactome for three RAMPs and 215 GPCRs representing all receptor subfamilies. All potential GPCR-RAMP interacting pairs were expressed ectopically, solubilized and analyzed using the multiplexed suspension bead array (SBA) strategy. In this assay, large panels of anti-GPCR and anti-RAMP antibodies (Abs) were used to capture and detect GPCR-RAMP complexes on color-coded magnetic microbeads in a flow-based format (*11*, *12*). The SBA assay detected GPCR-RAMP complexes with up to 11 different combinations of capture-detection pairs simultaneously. Our data showed that 122 of the GPCRs tested showed strong evidence for interaction with at least one RAMP. Most RAMP-interacting GPCRs formed complexes with either two or all three RAMPs. However, several GPCRs did not form complexes even when coexpressed with a RAMP. We used the SBA assay to show endogenous GPCR-RAMP complexes in three wild-type cell lines. We identified 23 GPCRs that formed complexes with at least one RAMP and validated the formation of several endogenous GPCR-RAMP2 complexes in situ by using a proximity-dependent assay in neuroepithelioma cells. We also conducted bioinformatic analyses of GPCR and RAMP expression in human cells and tissues and observed that RAMP2- and RAMP3-interacting GPCRs have similar RNA expression profiles. Most of the specific GPCR-RAMP interacting pairs we report in our study were previously unreported.

## RESULTS

### Workflow to map GPCR-RAMP interactions

We constructed an expression library of 215 dual epitope-tagged GPCRs (DuET library) (*12*) and an orthogonal library of three DuET RAMPs. All but four DuET GPCRs have an N-terminal FLAG and a C-terminal Rho1D4 epitope tag and were derived from the PRESTO-Tango GPCR library (*13*). Each RAMP has an N-terminal 3x hemagglutinin (3xHA) epitope tag and a C-terminal *E*. *coli* OmpF Linker and mouse Langerin fusion Sequence (OLLAS) epitope tag. We coexpressed the 215 DuET GPCRs pairwise with each RAMP in Expi293F cells. To facilitate multiplexing, we coupled anti-epitope tag monoclonal Abs (mAbs), anti-RAMP polyclonal Abs (pAbs), and 248 validated anti-GPCR pAbs primarily from the Human Protein Atlas (HPA) targeting 154 unique GPCRs (*12*) to color-coded magnetic beads. Then, we pooled the beads to create the SBA (table S1). We used six GPCR subfamily-specific SBA pools corresponding to the Glutamate, Rhodopsin, Adhesion, Frizzled/Taste2, Secretin (GRAFS) classification system: rhodopsin divided into alpha, beta, gamma, and delta; “other”; and glutamate, secretin, adhesion, and frizzled combined into one group.

**Fig. 1.**
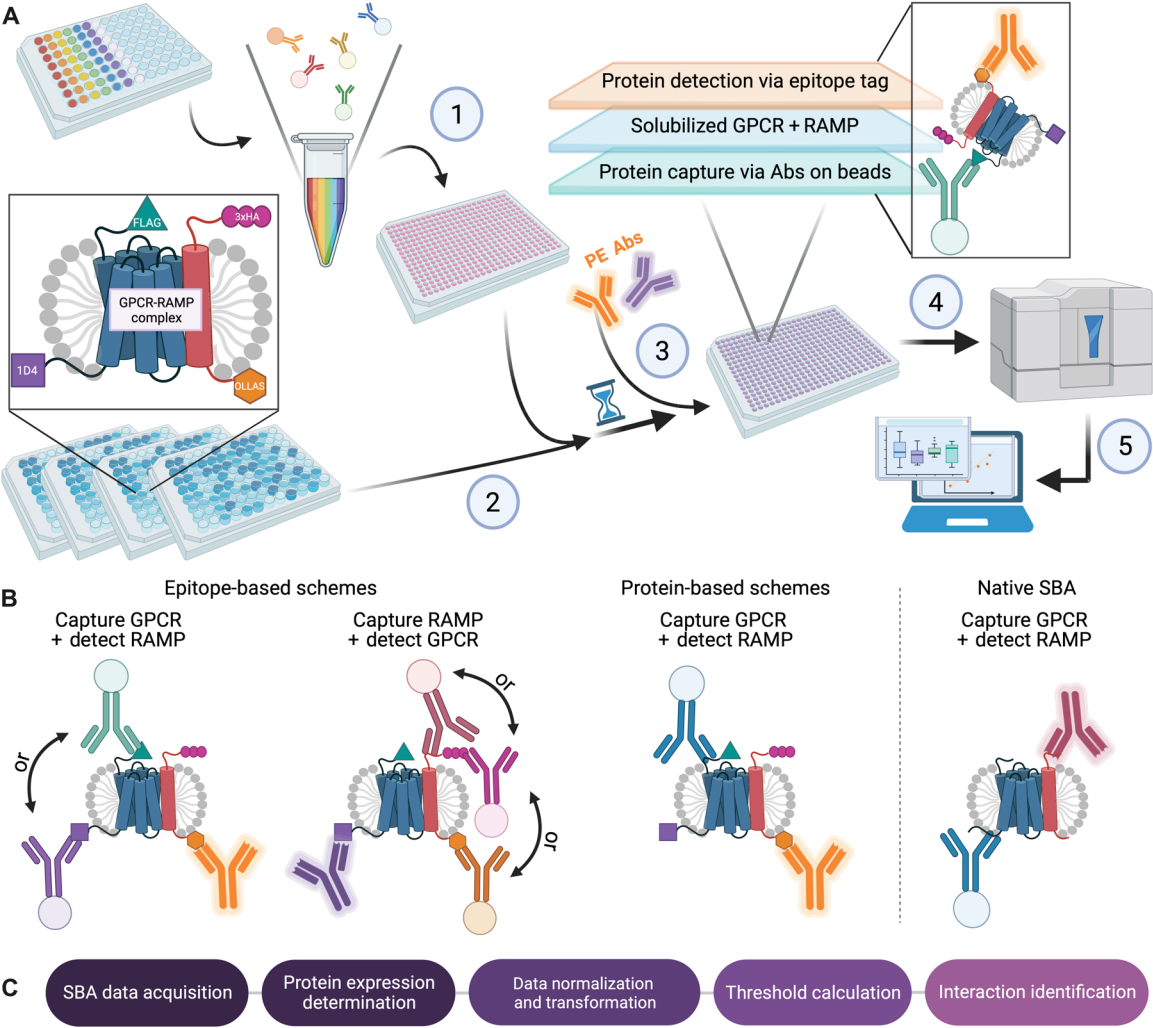
Multiplexed analysis of the GPCR-RAMP interactome using the DuET library. (**A**) An SBA assay experimental workflow was developed (*9*, *12*). Abs were grouped by the phylogenetic subfamily of their GPCR target and were coupled to unique color-coded beads and pooled to generate six subfamily-specific SBAs (*1*). A library of DuET GPCRs and RAMPs was expressed pairwise. Cells were solubilized to create heterogeneous mixtures of proteins, and concentrations were normalized across samples before incubation of aliquots with the SBAs (*2*). PE-conjugated anti-1D4 or anti-OLLAS mAbs were used to detect the GPCR-RAMP complexes captured by the Ab-coupled beads (*3*). The data were collected on a Luminex FlexMap three-dimensional (3D) instrument (*4*) and processed to identify GPCR-RAMP complexes. Results were integrated into an interactive web interface (*5*). (**B**) The GPCR-RAMP complex capture and detection schemes are shown schematically. In all cases, the reporter fluorescence produced by the PE-conjugated detection Ab was associated with the bar code of each bead. From a single well, GPCR-RAMP complexes could be detected simultaneously using anti-epitope tag Abs, anti-GPCR Abs, or anti-RAMP Abs. (**C**) Data analysis workflow. Data generated as described in (A) were first tested for GPCR or RAMP expression, then the fluorescence intensity data were normalized, and threshold values were calculated. Adapted from (*12*). Created in Biorender.com.

We applied the solubilized cell membrane samples containing the coexpressed libraries to the SBAs (Fig. 1A). We detected GPCR-RAMP complexes with either epitope-based or protein-based capture schemes (Fig. 1B). We generated data for epitope-based capture in five different assay design schemes: two schemes based on GPCR capture and three based on RAMP capture. In the assay, the GPCR was immunocaptured with anti-1D4 or FLAG mAbs, and then the GPCR-RAMP complex was detected via the RAMP using phycoerythrin (PE)–conjugated anti-OLLAS mAb. Similarly, bead-bound Abs captured the RAMP via anti-HA, anti-OLLAS, or anti-RAMP–specific Abs, and then the complex was detected via the GPCR using a PE anti-1D4 mAb. Using anti-GPCR pAbs, primarily obtained from the HPA project, the assay captured specific GPCRs and then used PE-conjugated anti-OLLAS mAbs targeting the RAMP to detect the presence of the bead-bound GPCR-RAMP complexes. To detect endogenous GPCR-RAMP interactions in untransfected cell lines, we captured GPCRs with anti-GPCR pAbs and detected the RAMP with PE-conjugated anti-RAMP pAbs. We selected SH-SY5Y cells, SK-N-MC cells, and Expi293F cells for the endogenous screen based on their reported RAMP expression profiles, RNA sequencing (RNA-seq) data, and accessibility (*2*, *14*–*16*) (proteinatlas.org).

To assess the SBA data systematically, we developed an interactome framework that was generalizable to both the heterologous and endogenous expression analysis, consistent across experiments and yet versatile enough to be customizable to different features of each dataset (Fig. 1C). After data collection, we subjected the reported median fluorescence intensity (MFI) levels to several quality control steps to ensure successful Ab-bead coupling to the beads and to quantify the amounts of solubilized protein added to the assay. We normalized the MFI values where appropriate for the analysis and transformed them into signal-to-noise ratios (SNRs), *Z*-scores, or robust *Z*-scores (R.*Z*-scores) for each capture-detection scheme. Next, we annotated all potential interactions by setting thresholds for each capture-detection scheme for each RAMP based on existing knowledge. To enable interactive, transparent, and user-friendly access to the data, we developed a web-based open-access interface (Shiny App, fig. S1, accessible at https://leod.shinyapps.io/gpcr_ramp_interactome). The interface displays information about the different data layers reported per GPCR-RAMP interaction, summarizes the interactome analysis for each GPCR or across subfamilies, and allows for browsing in a GPCR-centric manner.

### Exploring the GPCR-RAMP interactome

#### Validation of constructs, controls, and SBA assay

To evaluate the suitability of the DuET library for the SBA assay, we used CALCRL in complex with each of the three RAMPs as positive controls and measured agonist-dependent inositol monophosphate (IP1) accumulation using the promiscuous Gqs5 in a homogeneous time-resolved fluorescence (HTRF) assay (fig. S2A and table S2). Cells coexpressing the DuET CALCRL (FLAG-CALCRL-1D4) construct and appropriate RAMP were treated with calcitonin gene related peptide (CGRP) or adrenomedullin (AM). The IP1 accumulation responses elicited by these CALCRL-RAMP pairs were compared with cells coexpressing the HA-CALCRL-1D4 construct with each RAMP as used earlier (*9*). We found that the 3xHA- and OLLAS-DuET RAMPs were equally capable of forming functional CALCRL-RAMP complexes compared with the FLAG- and OLLAS-DuET RAMPs used earlier (*9*). All the CALCRL-RAMP1 complexes tested signaled in response to CGRP and AM stimulation, and all the CALCRL-RAMP2 and CALCRL-RAMP3 complexes tested signaled in response to AM stimulation. This confirms prior knowledge about the CALCRL interactome.

Next, we evaluated the ability of the SBA assay to detect GPCR-RAMP complexes (fig. S2B). We generated solubilized cell membrane samples using the DuET CALCRL construct (FLAG-CALCRL-1D4) coexpressed with either 3xHA-RAMP3-OLLAS or FLAG-RAMP3-OLLAS (*9*). We then captured CALCRL-RAMP3 using an SBA composed of anti-epitope tag, anti-CALCRL, or anti-RAMP3 Abs bound to beads. PE-labeled anti-1D4 or anti-OLLAS mAbs were used to detect the captured complexes (fig. S2B). The two RAMP3 constructs exhibited similar expression, as determined by anti-RAMP3–specific capture and OLLAS detection. Both constructs showed concordant ability to form CALCRL-RAMP3 complexes when coexpressed with CALCRL. The complex was consistently detected across five of the six capture-detection schemes tested.

To evaluate further the FLAG-CALCRL-1D4 construct, we generated solubilized membrane samples from cells coexpressing either the DuET CALCRL construct or the HA-CALCRL-1D4 construct used earlier (*9*) with 3xHA-RAMP3-OLLAS (fig. S2C). After subjecting these samples to the SBA assay, we saw similar levels of relative CALCRL protein expression and CALCRL-RAMP3 complex formation. Notably, 1D4- and OLLAS-based capture performed better than FLAG- and HA-based capture approaches. Together, these results confirm the functionality of the DuET RAMP1, RAMP2, and RAMP3 constructs and the robustness of the SBA assay to capture the positive control CALCRL-RAMP3 complex.

To judge the statistical reproducibility of SBA assay measurements, we expressed two GPCRs from different subfamilies with or without a RAMP in biological triplicate. We then performed the SBA assay in technical duplicate (fig. S3 and table S3). GPCR class C group 5 member A (GPRC5A) was expressed with or without RAMP2 (fig. S3A), and orexin receptor type 2 (HCRTR2) was expressed with or without RAMP3. Four parallel epitope-based capture-detection strategies were used to detect the complexes (fig. S3B). Anti-HA or anti-OLLAS mAbs were used to capture the RAMP, while PE-conjugated anti-1D4 mAb was used to detect the GPCR in the complex. Conversely, anti-1D4 or anti-FLAG mAbs were used to capture the GPCR, while PE-conjugated anti-OLLAS mAb was used to detect the RAMP in the complex. The one-sided unpaired Wilcoxon test confirmed a statistically significant difference between the MFI levels from the coexpressed GPCR-RAMP and GPCR-mock samples. The GPRC5A-RAMP2 and HCRTR2-RAMP3 complexes have not been reported earlier.

The results described above confirm the suitability and reliability of the multiplexed SBA assay to identify GPCR-RAMP complexes in the setting of a validated positive control using both technical and biological replicates. For the DuET library–based GPCR-RAMP interactome screen, we used one biological replicate of each of the four unique GPCR-containing samples (each GPCR alone and each GPCR with each of the three RAMPs) in two replicates. Each replicate represented one detection scheme. We analyzed 860 solubilized cell membrane samples along with controls, which corresponded to six 384-well SBA assay plates, to generate approximately 40,000 unique data points.

To determine the expression levels of each RAMP, we used the MFI values arising from capturing the HA tag on the RAMP or capturing the native RAMP sequence and detecting the OLLAS tag (fig. S4A and table S3). We confirmed significantly elevated expression levels for all three RAMPs (*P* < 0.0001) and observed that each anti-RAMP Ab was specific for its intended RAMP target. A similar strategy was previously used to measure the expression of the GPCRs (*12*). The positive control, CALCRL-RAMP1, was evaluated in more detail. The positive control complex was analyzed over 10, 11, 20, or 22 technical replicates depending on the capture-detection scheme. The complex showed highly significant expression levels and complex formation (*P* < 0.0001) in all combinations when compared with mock across all capture-detection schemes (fig. S4, B and C).

#### Detection of GPCR-RAMP complexes using engineered epitope tags

**Fig. 2.**
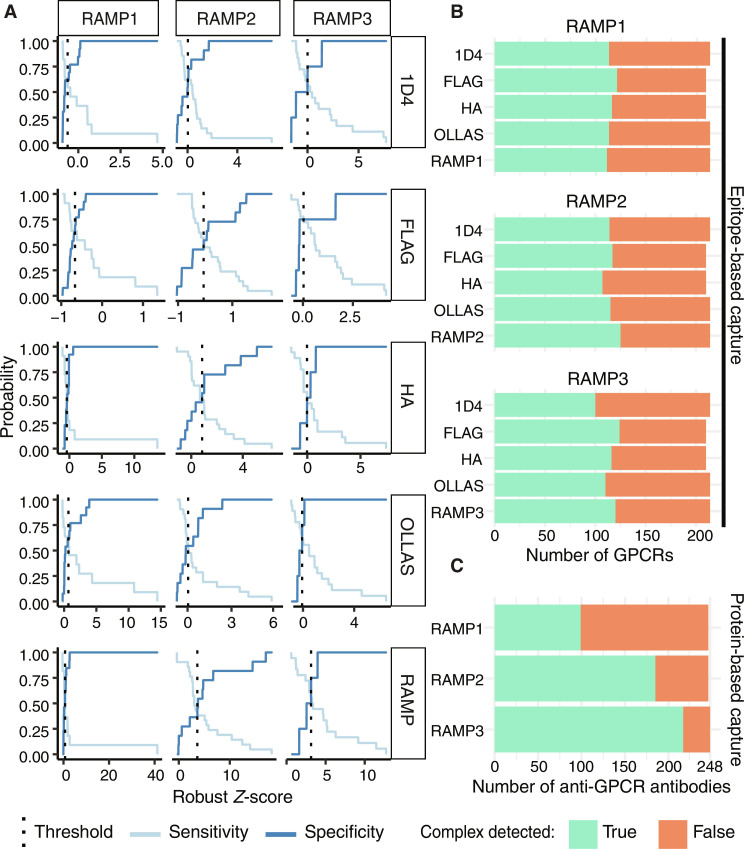
Identification of GPCR-RAMP complexes. (**A**) Thresholds for each RAMP and epitope-based capture-detection scheme were chosen as the Robust *Z*-score (R.*Z*-score) where the sensitivity (light blue) and selectivity (dark blue) curves intersect for GPCR-RAMP interactions known from the literature (*1*). The boxed label on the right of each row indicates the capture scheme. Anti-1D4 and anti-FLAG capture (GPCR capture) corresponds to anti-OLLAS RAMP detection. Anti-HA, anti-OLLAS, and anti-RAMP capture correspond to anti-1D4 GPCR detection. (**B**) The thresholds determined in (A) and in table S6 were applied to identify GPCR-RAMP interactions. Summary plots of the total number of GPCR-RAMP interactions detected for 215 GPCRs, per RAMP and capture-detection scheme. (**C**) Summary plot of the total number of GPCR-RAMP interactions detected, per RAMP, using protein-based capture with 248 anti-GPCR Abs corresponding to 154 unique GPCRs. Green, complex detected; orange, complex not detected.

To map the GPCR-RAMP interactome for the 215 receptors tested with the epitope-based capture approach, we computed a threshold for each capture-detection scheme to assign interactions as “hits” for each RAMP (Fig. 2A and table S4) by creating a model based on interacting and noninteracting pairs reported in the literature (*1*). We calculated the true positive (TP), true negative (TN), false positive (FP), and false negative (FN) values at different thresholds to generate sensitivity and specificity curves and determined the threshold at the intersection point of the curves (table S4). The results from applying these thresholds are presented as a binary heatmap for each capture-detection scheme (fig. S5A). They can be summed across each column to determine the total number of GPCRs that interact with each RAMP in each capture-detection scheme (Fig. 2B). The number of TP, TN, FP, and FNs for each RAMP in each capture-detection scheme is listed in table S5. Dissimilarities in the thresholds observed between different capture-detection schemes can be explained by experimental differences in binding affinities of the Abs and differences in the published GPCR interactions reported for each RAMP. Overall, there is agreement across all capture-detection schemes in the total number of RAMP-interacting GPCRs identified: 54% of GPCRs tested had measurable complex formation with each RAMP across all five capture-detection schemes. Twenty-three GPCRs formed complexes with all three RAMPs detected by all five capture-detection schemes. Only nine GPCRs did not form any detectable complexes with at least one of the RAMPs in any capture-detection scheme.

#### Detection of GPCR-RAMP complexes using validated anti-GPCR Abs

We used 248 anti-GPCR Abs that recognize 154 unique GPCRs to generate additional evidence for potential GPCR-RAMP interacting pairs (*12*). The protein-based capture approach does not require GPCRs with engineered epitope tags. We selected Ab-specific interaction thresholds (table S6) using a population density–based approach on the R.*Z*-scores, analogous to that described in the context of the previous Ab validation study (*12*). We selected a stringency of six median absolute deviations (MADs) above the data population density peak. Consistent with the results from epitope-based capture, widespread GPCR-RAMP interactions were detected among the 154 GPCRs tested, with an overall hit rate of approximately two-thirds (fig. S5B and table S5). RAMP1 exhibited the lowest frequency of interactions, where 99 of 248 Abs (40.0%) captured 74 unique GPCR-RAMP1 complexes. Conversely, we detected 128 GPCR-RAMP2 complexes, corresponding to 185 unique capture Abs (74.6%), and 139 GPCR-RAMP3 complexes, corresponding to 217 unique capture Abs (87.5%).

#### Comparison of GPCR-RAMP complex detection schemes

We compared the overall results from epitope-based and protein-based capture schemes. First, we assessed whether any capture-detection schemes were subject to bias caused by relative GPCR or RAMP expression levels. On the basis of the proportion of capture-detection schemes (epitope-based and protein-based capture considered separately) with GPCR-RAMP interactions detected, GPCR-RAMP pairs were classified into three groups of interaction evidence: weak (<33%), medium (>33% and <67%), and strong (>67%). We then examined the GPCR-RAMP interaction hits distribution for each GPCR or RAMP expression quartile (figs. S6 and S7). We did not observe any patterns of interaction evidence correlating with RAMP expression levels (figs. S6, A and B, and S7A), indicating that the RAMP expression levels did not bias the complex detection results. We observed fewer high-confidence interactions for RAMP1 in the protein-based capture format, and RAMP1 generally expresses less efficiently than RAMP2 or RAMP3. There was a slight tendency of expression bias in the RAMP2 dataset for the protein-based capture format. In contrast, we observed some bias in the distribution of hits across GPCR expression quartiles for the epitope-based but not the protein-based capture formats used to detect GPCR-RAMP complexes (fig. S6, C and D). Parsing the epitope-based data into individual capture-detection schemes reveals that the cascade distribution can be attributed to capture-detection formats that capture the RAMP (HA, OLLAS, or RAMP-specific capture) and detect the GPCR (1D4 tag detection) (fig. S7B). This pattern suggests that the GPCR may be limiting in the assay format when it is used for detection.

**Fig. 3.**
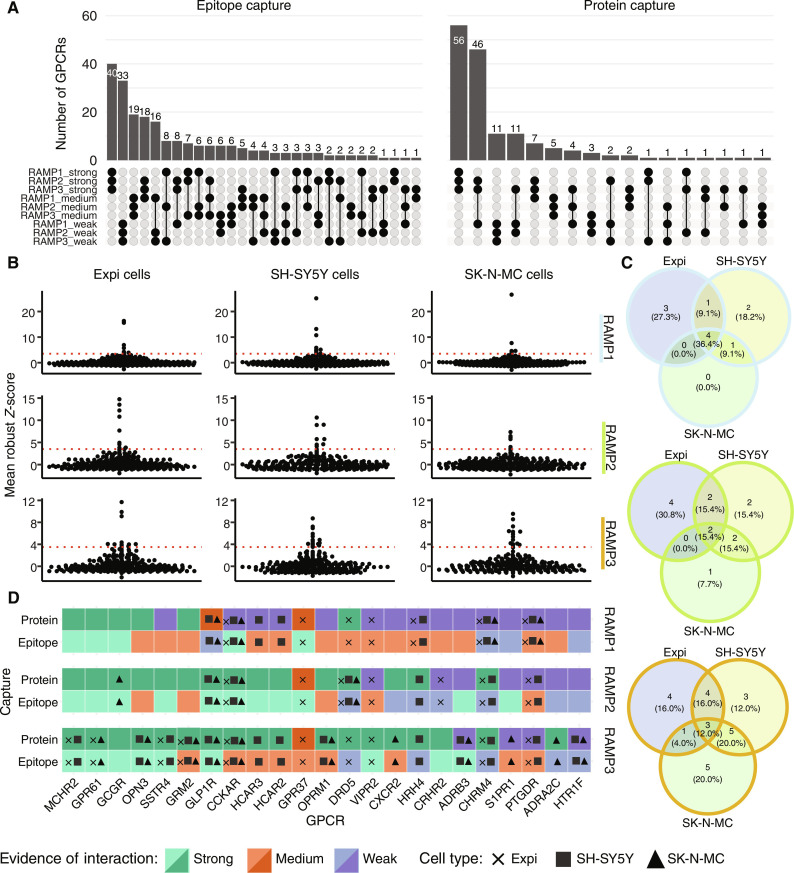
Detection of GPCR-RAMP complexes in transfected and wild-type cell lines. (**A**) UpSet plots show the overlap between the sets of GPCRs in the DuET library that interacted with RAMP1, RAMP2, and RAMP3 based on epitope capture (left) or protein capture (right). There were five unique epitope-based capture-detection schemes and between zero and six unique protein-based capture-detection schemes for each GPCR-RAMP pair studied. Protein capture data were generated for 154 GPCRs (of 215) for which validated anti-GPCR Abs were available. (**B**) The SBA assay (242 Ab-coupled beads against 148 unique GPCRs) was used to analyze solubilized membranes from Expi293F (Expi), SH-SY5Y, and SK-N-MC cells (*12*). Endogenous GPCR-RAMP complexes were detected for each cell line with anti-GPCR capture and anti-RAMP1 (top), anti-RAMP2 (middle), or anti-RAMP3 (bottom) detection. Data are plotted as the mean R.*Z*-score of the replicates measured for each anti-RAMP detection Ab. The GPCR Abs with scores above threshold of 3.5 (red dotted line) are listed in table S8. (**C**) Venn diagrams for anti-GPCR Ab hits across the three cell lines tested for GPCR interaction with RAMP1 (light blue outline), RAMP2 (green outline), and RAMP3 (orange outline). Data are from biological triplicates measured in technical duplicate for each detection scheme. (**D**) Heatmap results for GPCR-RAMP endogenous interactome screen in Expi cells (x), SH-SY5Y cells (filled square), and SK-N-MC cells (filled triangle). Strong, >66% passing capture-detection schemes; medium, 33 to 66% passing capture-detection schemes; weak, <33% passing capture-detection schemes.

To determine whether RAMP-interacting GPCRs tended to interact with only one RAMP, all three RAMPs, or a subset of the RAMPs, we investigated the overlap between the results for epitope-based and protein-based capture schemes (Fig. 3A). For both datasets, the largest group of GPCRs were those with strong evidence for interactions with all three RAMPs—40 GPCRs in the epitope-based capture set and 56 in the protein-based capture set. There were 20 GPCRs with strong evidence for interaction with all three RAMPs across both datasets. The epitope-based capture results reveal that GPCRs that do not interact with all three RAMPs were next most likely to interact with none of the RAMPs (33 GPCRs). The protein-based capture results show that GPCRs interacting with two or fewer RAMPs were most likely to interact with RAMP2 and RAMP3 (46 GPCRs). Notably, only 17 GPCRs showed strong evidence for complex formation with a single RAMP.

RAMP interaction partners for each GPCR are presented in table S7. Nine GPCRs showed evidence for complex formation with all three RAMPs across all capture-detection methods: GABBR1, GPR143, GPR21, GPR61, HTR4, LPAR2, MTNR1A, OXER1, and P2RY11 (see table S1 for the corresponding GPCR UniProt IDs). Considering each RAMP individually, 24 GPCRs showed strong evidence for interaction with RAMP1 across all capture-detection methods, 37 GPCRs showed strong evidence for interaction with RAMP2, and 34 GPCRs showed strong evidence for interaction with RAMP3. The intersection of these three sets reveals 58 GPCRs, the number of unique GPCRs with positive interaction evidence in every epitope-based and protein-based capture-detection format for interaction with at least one RAMP. Only seven of these GPCRs have been previously tested for complex formation with the RAMPs (*1*). Overall, there was satisfactory agreement between the results from epitope-based and protein-based capture of GPCR-RAMP complexes.

### Detection of endogenous GPCR-RAMP complexes

Following the SBA screen with the engineered DuET library, we applied the SBA assay to detect endogenous GPCR-RAMP interactions in solubilized membranes from Expi293T, SK-N-MC, and SH-SY5Y cell lines. In addition to Expi293F cells, which were used in the original SBA screen, the neuroepithelioma-derived SK-N-MC cell line was used because it has been used as a reference cell line for the discovery of RAMPs, and the SH-SY5Y cell line was used for comparison as a related neuroblastoma cell line. We included Expi293F cells transfected with CALCRL alone or cotransfected with each of the three RAMPs to confirm the functionality of the experiment (fig. S8 and table S3). We used two validated anti-CALCRL Abs for capture and PE-conjugated anti-OLLAS mAb, anti-RAMP1 pAb, anti-RAMP2 pAb, and anti-RAMP3 pAb for detection of ectopically expressed CALCRL-RAMP complexes. As expected, we detected all three CALCRL-RAMP complexes with the CALCRL-OLLAS capture-detection scheme. We detected the correct CALCRL-RAMP complex with CALCRL capture and RAMP-specific detection at high statistical significance compared with the MFI levels from samples derived from cells with CALCRL expressed alone. There were differences in the performances of the schemes used for RAMP detection. A comparison of the SNRs for positive and negative samples within each detection scheme showed that RAMP3 detection performed better than RAMP2 detection, which, in turn, performed better than RAMP1 detection. The difference in detection Ab performance may be attributed to different affinities of each anti-RAMP Ab, different relative levels of ectopic expression of each RAMP, or a combination of the two factors.

Next, we validated endogenous RAMP expression in the three cell lines using anti-RAMP pAbs for both capture and detection (fig. S9 and table S3). Three of the five anti-RAMP pAbs used for capture were the same as those used for detection, and two of the anti-RAMP pAbs used for capture were distinct from those used for detection. For the three identical capture-detection anti-RAMP pAbs, each was raised against immunogens of 87 to 103 amino acids in length. Therefore, we reasoned that the same pAb could be used for capture and detection, as individual Abs typically recognize epitopes of five to seven amino acids (*17*). However, the structural representation of epitopes also affects Ab recognition, so the results must be interpreted carefully (*18*). Two Abs used for capturing RAMP1 or RAMP2 were anti-RAMP pAbs from the HPA. We found a range of endogenous RAMP expression levels that reached statistical significance compared to the negative control (buffer only). Overall, SK-N-MC and SH-SY5Y cells exhibited higher protein expression levels of a given RAMP than Expi293F cells.

We next mapped the endogenous GPCR-RAMP interactome in the three wild-type cell lines. We found 11, 13, and 25 unique GPCR-RAMP complexes for RAMP1, RAMP2, and RAMP3, respectively (Fig. 3, B and C, and table S8). Two anti–cholecystokinin A receptor (CCKAR) Abs captured endogenous CCKAR-RAMP1 and CCKAR-RAMP3 complexes, and two different anti–dopamine receptor D5 (DRD5) Abs captured endogenous DRD5-RAMP2 interactions. In both cases, one of the GPCR-specific Abs captured the GPCR-RAMP complex in all three cell lines, while the second Ab targeting the same GPCR captured the same GPCR-RAMP pair in only one or two of the cell lines. This observation may be explained by different Ab affinity toward the endogenous receptors and different expression levels of GPCRs per cell line. Three GPCRs were common among the three cell lines for interactions with RAMP1 or RAMP3, and two GPCRs were shared between all cell lines for interactions with RAMP2 (Fig. 3D). Overall, the most significant number of GPCR interactions was identified for RAMP3, and of those GPCRs, many were also identified as RAMP3-interacting hits in our GPCR-RAMP interactome screen (76%, protein-based capture; 33%, epitope-based capture).

The screening of endogenous GPCR-RAMP interactomes in cell lines is inherently limited because different lines express different repertoires of GPCRs. Cells typically express at least 100 GPCRs, including orphan receptors, which are often expressed at high levels (*19*). Future work screening tissue samples and cell lines derived from different tissues will likely reveal additional endogenous GPCR-RAMP interactions that we did not detect here. For example, the adenosine A1 receptor showed strong evidence for RAMP interaction by the SBA assay screen, but it was not identified in the GPCR-RAMP cell–based interactome assay.

### Detection of endogenous GPCR-RAMP complexes in cell membranes

To test for GPCR-RAMP complexes in endogenous cell membranes without heterologous overexpression and membrane solubilization, we used a proximity-based assay called the MolBoolean method (*20*). The assay allowed us to quantify selected GPCR-RAMP2 complexes in SK-N-MC cells relative to total GPCR and RAMP2 levels for each unique receptor. The MolBoolean method is based on the proximity ligation assay (PLA) concept. It generates rolling circle amplification products (RCPs) to localize a fluorescence signal in situ in a cell membrane environment. However, unlike the PLA, the MolBoolean method enables simultaneous visualization of individual proteins and those forming a complex and is well suited to provide evidence for the physiological relevance of GPCR-RAMP interactions identified by SBA assay.

We first used multiple controls to verify that the endogenous interactions between CALCRL and all three RAMPs could be detected by the MolBoolean assay (fig. S10 and table S3). Omitting individual primary Abs before sample processing enabled us to measure nonspecific binding of both primary Abs and MolBoolean probes, which, in turn, allowed us to determine the specific source of any background noise (fig. S10 and table S3). The number of RCPs per cell was significantly higher in cells incubated with anti-CALCRL and anti-RAMP primary Abs than in cells that received control treatments. As expected, we observed that many of the puncta corresponding to each RAMP were not in complex with CALCRL, which is consistent with the expectation that RAMPs have many GPCR-interacting partners in a typical cell membrane.

**Fig. 4.**
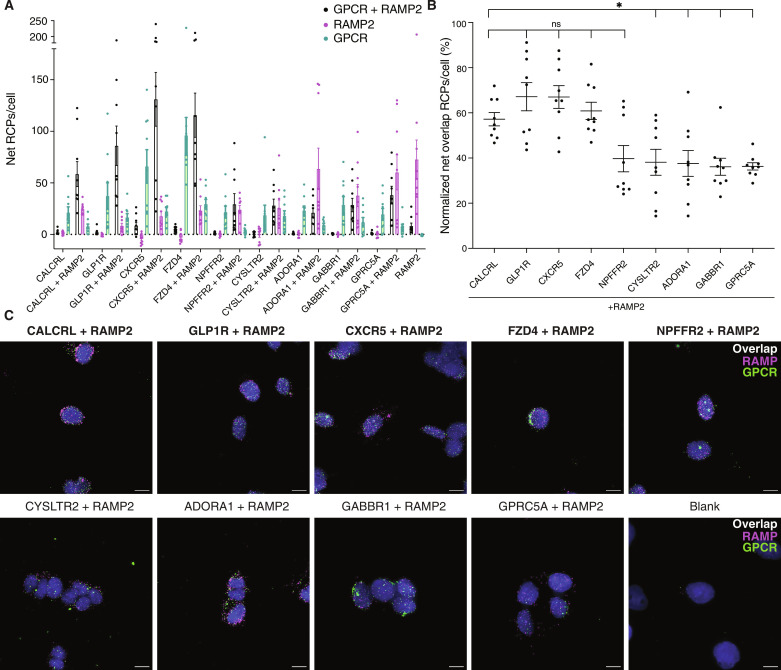
Endogenous GPCR-RAMP2 complexes in SK-N-MC cell membranes. To quantitate GPCR-RAMP2 interactions, the number of MolBoolean RCPs per cell for each Z-stack captured from a different field of view was measured. (**A**) The complexed and uncomplexed GPCR and RAMP2 are quantified as the number of net RCPs per cell for cells stained for the specified GPCR only or for the GPCR and RAMP2. (**B**) For each GPCR-RAMP2 pair, the percentage of RCPs out of the total number of RCPs per cell is quantified. Statistical significance was determined by one-way analysis of variance (ANOVA) followed by Dunnett’s multiple comparisons test to the positive control, CACLRL-RAMP2 (*P < 0.05). ns, not significant. Sample sizes and *P* values are listed in table S3. (**C**) Representative Z-stack maximum projection images of cells subject to MolBoolean assay. Scale bars, 10 μm. Blue, 4′,6-diamidino-2-phenylindole (DAPI); green, GPCR puncta; magenta, RAMP2 puncta; white, GPCR-RAMP2 complex puncta. RCPs from MolBoolean-stained cells that were not incubated with any primary Ab were subtracted to obtain net RCP values. Labels on the *x* axis in (A) and (B) and on top of the images in (C) indicate the target(s) of the included Abs. The positive control and samples that did not differ significantly from it are bolded. Data are from three biological replicates performed with at least three technical replicates.

We next used the MolBoolean method to test endogenous GPCR-RAMP2 interactions in SK-N-MC cells for eight GPCRs included in the SBA assay screen. We stained SK-N-MC cells with Abs targeting each GPCR or costained with an Ab targeting each GPCR and an Ab targeting RAMP2. We stained for CALCRL and RAMP2 as the positive control. Overall, the GPCRs were found to interact with RAMP2 (Fig. 4 and tables S1 and S3). Cells stained for RAMP2 and four of the GPCRs tested exhibited a percentage of overlap RCPs per cell (RCPs corresponding to GPCR-RAMP complexes) that did not differ significantly from the positive control. Although the remaining GPCR-RAMP2 pairs exhibited a statistically significant difference in overlap RCPs per cell compared to CALCRL-RAMP2, complex formation was still observed.

### Bioinformatic analysis of the GPCR-RAMP interactome

We carried out a bioinformatic analysis to investigate potential implications of the GPCR-RAMP complexes identified experimentally. We first created a matrix to integrate both epitope-based and protein-based results from the SBA assay results. Each GPCR studied was assigned as “yes,” “no,” or “inconclusive” to indicate its ability to interact with each RAMP. Of the 215 unique GPCRs, 205 were annotated as either yes or no (fig. S11A). Notably, 51 GPCRs interacted with all three RAMPs, and 45 GPCRs interacted with both RAMP2 and RAMP3. A smaller number of GPCRs interacted with only a single RAMP. There were 24 GPCRs that did not interact with any RAMP, and 49 GPCRs did not interact specifically with RAMP1.

We used the RNA expression levels of the GPCRs in human cell types from single-cell RNA-seq data hosted by proteinatlas.org (*21*, *22*). We defined expression as normalized transcripts per million (nTPM) > 1 per cell type and only considered cell types in which corresponding RAMP transcripts were coexpressed (fig. S11A). We then calculated the percentage of expected RAMP-interacting (yes) or non-RAMP–interacting (no) GPCRs for each RAMP. The number of GPCRs expressed varied between 18 and 71 across different cell types. For each RAMP, the percentage of yes and no interaction GPCRs is displayed for each cell type (fig. S11B).

We next examined for each RAMP whether the GPCR:RAMP expression ratios for GPCR-RAMP pairs in single cells differed for GPCRs annotated as yes compared with those annotated as no (fig. S12A). We observed significantly higher expression ratios (*P* < 0.005, Wilcoxon test; table S3) for the GPCRs expected to interact with RAMP3 compared with those that did not interact with RAMP3. These results indicate that in a given cell type, RAMP3-interacting GPCRs are more likely to be expressed at a higher ratio compared with RAMP3 levels than GPCRs within the same cell types that do not interact with RAMP3. In contrast, no significant differences in expression ratio were identified for interacting and noninteracting GPCR-RAMP1 or GPCR-RAMP2 pairs.

To contextualize our analysis of single-cell GPCR and RAMP expression levels, we investigated RAMP expression in different human tissues and individual cell types using data hosted by the HPA (proteinatlas.org; fig. S12, B and C) (*21*, *22*). RAMPs are ubiquitously expressed in human tissue, except for bone marrow (no RAMP expression) and the retina (RAMP2 expression only; fig. S12B). However, RAMP expression is much less homogeneous when examined at the single-cell level, which is important to understand for determining the physiological effects of each RAMP. RAMP2 and RAMP3 expression patterns (nTPM > 1) are more similar than other RAMP pairwise combinations. We applied hierarchical clustering to the single-cell data, confirming that RAMP2 and RAMP3 were clustered, indicating more similar expression profiles (fig. S12C).

Last, we tested whether GPCR-RAMP interaction correlated with distinct downstream GPCR signaling partners. We calculated the distribution of the different G protein and β-arrestin (βArr) couplings for GPCRs that interact with at least one RAMP compared with GPCRs that do not interact with any RAMP using coupling information from GPCRdb.org (fig. S13) (*23*–*26*). G protein coupling information was available for 90 GPCRs, and 32 of these GPCRs also had information available on βArr coupling. We did not observe any statistically significant differences between the coupling distributions for specific GPCR-RAMP interacting pairs, except when comparing secondary G protein coupling types (*P* value < 0.05, Fisher exact test; table S3). RAMP-interacting GPCRs are more likely to have a secondary coupling to Gs rather than to Gq/11. In addition, most GPCRs that couple with G12/13 as a secondary G protein do not interact with RAMPs.

## DISCUSSION

We used a customized combinatorial library and a bead-based multiplexed screening platform to elucidate the interactome between 215 GPCRs and three RAMPs. The results provide strong experimental evidence to support the hypothesis that GPCR-RAMP interactions are widespread across all phylogenetic GPCR subfamilies. Most GPCRs tested interacted with at least one RAMP, and approximately one-quarter of the GPCRs interacted with all three RAMPs. Several GPCR-RAMP interactions detected in the interactome screen were also classified as hits in the endogenous interactome screen.

Glucagon-like peptide 1 receptor (GLP1R)–RAMP2 complexes were robustly detected in the SBA assay and in cell membranes by the MolBoolean proximity assay. The agreement across assays underscores the robustness of the screening strategy. GLP1R has previously been reported to interact with the RAMPs based on studies conducted only in heterologous overexpression systems (*9*, *27*, *28*). The in vivo effects of RAMP1 and RAMP3 double knockdown on the activity of different peptides that target GLP1R and the glucagon receptor (GCGR) have recently been reported (*29*). We show that solubilized, endogenous GLP1R forms complexes with all three RAMPs in SK-N-MC and SH-SY5Y cells and that there are endogenous GLP1R-RAMP2 complexes in membranes in SK-N-MC cells. These results may have important implications for GLP1R pharmacology and the design of therapeutics for metabolic and autoimmune diseases. GLP1R has recently been implicated as a multimodal receptor involved in cardiometabolic disease and is targeted by several US Food and Drug Administration–approved drugs for the treatment of type 2 diabetes and obesity (*30*–*32*).

Another example is the orphan receptor GPRC5A, which formed complexes with RAMP2 and RAMP3 as detected by the SBA assay with strong evidence across both epitope-based and protein-based capture approaches. The presence of GPRC5A-RAMP2 complexes in cell membranes was confirmed by the MolBoolean method. GPRC5A is reported to play a tumor-suppressive role, and its down-regulation has been implicated in lung, pancreatic, colorectal, and breast cancer pathology, although its endogenous ligand remains unknown (*33*–*35*). Future investigations of GPRC5A in the presence of RAMP may lead to its successful deorphanization. Notably, the other three members of the “GPCR family C group 5” subfamily (GPRC5B, GPRC5C, and GPRC5D) also exhibited evidence for complex formation with RAMP2 and RAMP3. Follow-up studies may reveal subtype-specific modes of regulation of the different receptors by the RAMPs.

Bioinformatic analyses of single-cell RNA-seq data provide additional insights concerning the GPCR-RAMP interactome. Independent experimental SBA assay data and single-cell RNA-seq data were available for 205 GPCRs, a scale not previously reported. The SBA screen suggests that many GPCRs interact with both RAMP2 and RAMP3 but not RAMP1. This experimental observation is supported in the bioinformatic investigation. For example, in cells that contain both RAMP-interacting and noninteracting GPCRs, RAMP2- and RAMP3-interacting GPCRs exhibit similar expression profiles. Cellular RAMP2 and RAMP3 expression patterns cluster and are distinct from RAMP1 expression patterns. Although RAMP3 is expressed in the fewest distinct cell types, it is also the most likely RAMP to interact with GPCRs. On the basis of this result and the observation that the GPCR:RAMP expression ratio is significantly higher for RAMP3-interacting GPCRs than for noninteracting GPCRs, it follows that RAMP3 is the most promiscuous of the three RAMPs.

Bioinformatic analysis of GPCR coupling to different G proteins and βArr was used to gauge potential functional consequences of GPCR-RAMP complex formation. The results suggest that RAMP interaction correlates with a different secondary G protein coupling profile for a given GPCR. This effect may be caused by a change in cellular localization promoted by RAMP association. However, the number of GPCRs with available information was low, especially for secondary G protein couplings, and further investigation is necessary.

In summary, an affinity proteomic approach was used to detect GPCR-RAMP complexes in solubilized membranes from cells heterologously expressing GPCRs and RAMPs and from cell lines endogenously expressing GPCRs and RAMPs. Several GPCR-RAMP complexes were further investigated in cell membranes in situ using a proximity assay. At least 50 previously unidentified GPCR-RAMP complexes were identified. Overall, the data strongly suggest the widespread occurrence of GPCR-RAMP complexes among at least one-half of the GPCRs tested. The methodology presented is scalable and flexible and can be readily adapted for basic and translational applications, such as detecting GPCR heterodimers, interactions with regulatory proteins, and screening for pathological anti-GPCR autoantibodies.

## MATERIALS AND METHODS

### RAMP constructs

Epitope-tagged, codon-optimized human RAMP1, RAMP2, and RAMP3 constructs were encoded in a pcDNA3.1(+) expression vector. The plasmids encode an N-terminal 3xHA tag (amino acid sequence YPYDVPDYA) following the signal peptide (SP; amino acids 1 to 26, 1 to 42, and 1 to 27 for RAMP1, RAMP2, and RAMP3, respectively) and two C-terminal OLLAS tags (amino acid sequence SGFANELGPRLMGK) linked by a flexible spacer that also includes two Strep-tag II peptides (amino acid sequence WSHPQFEKGGGSGGGSGGGSWSHPQFEK). Each 3xHA-RAMP-OLLAS construct was generated using the TagMaster site-directed mutagenesis kit according to the manufacturer’s instructions for “long-range mutation” as previously reported (*36*) using the previously validated FLAG-RAMP-OLLAS construct as a starting point (*9*). All constructs were confirmed by sequencing in the forward and reverse directions (T7, BGHR primers) (Genewiz).

### DuET library of GPCR constructs for GPCR-RAMP interactome analysis

The DuET library was generated as described in Dahl *et al.* (*12*) and is available on Addgene. Briefly, epitope-tagged human GPCR DNA constructs were encoded in pcDNA3.1(+). All GPCRs except CALCRL and Frizzled-4 (FZD4), FZD5, FZD6, and FZD10 were generated on the basis of the PRESTO-tango library of signal sequence-FLAG-GPCRs as a starting point. The PRESTO-Tango plasmid kit was a gift from B. Roth (Addgene kit #1000000068). The DuET FLAG-GPCR-1D4 constructs encode the FLAG tag (DYKDDDDA) following the HA SP, MKTIIALSYIFCLVFA. C-terminal components of the original PRESTO-Tango constructs were removed (V2 tail, Tobacco Etch Virus (TEV) site, Tta transcription factor) upon the addition of a full-length 1D4 tag (DEASTTVSKTETSQVAPA). The forward primer used for all receptor was CGTTTAAACTTAAGCTTAGCGCCACCATGAAGACGATCATC (5′ to 3′), and the reverse primer used was TGCTGGCCTCATCGAATTCACCGGTGCGTCCACCGGTATC (5′ to 3′). The primers were designed using the NEBuilder Assembly Tool on the New England Biolabs (NEB) website and purchased from Integrated DNA Technologies at the standard desalting grade. The pcDNA3.1(+) vector encoding the C-terminal 1D4 tag was subject to enzymatic double digestion and purification. FLAG-CALCRL-1D4 with its endogenous SP removed was generated by replacing the HA tag with a FLAG tag in a previously validated FLAG-CALCRL-1D4 construct (*9*). As described earlier, during the construction of the PRESTO-Tango library, 23 GPCRs with an endogenous SP within the coding sequence ended up with a second upstream HA SP in the final plasmid. Therefore, we removed the endogenous SP from 12 GPCRs (F2R, GABBR1, GLP1R, GPR156, GPR37, GPR97, GRM1, GRM2, GRM4, GRM5, GRM6, and GRM7) but not from 11 others (CALCR, CD97, CRHR1, CRHR2, GCGR, GIPR, GPR114, GPRC5B, GPRC5C, GPRC6A, and VIPR2) due to financial and time considerations. There were no FZD receptors in the PRESTO-tango library, so we designed synthetic genes for FZD4, FZD5, FZD6, and FZD10, which were synthesized at Genewiz. The human FZD4, FZD5, FZD6, and FZD10 plasmids encode the 5-HT3a SP in place of the endogenous SP, followed by an HA epitope tag. We introduced a codon for Ala instead of the codon for Arg to optimize the Kozak sequence (GCCGCCACCATGG). The C-terminal tail of the engineered receptors was the full-length (18 amino acid) 1D4 mAb epitope tag (*3*). All constructs were confirmed by sequencing in the forward and reverse directions (T7, BGHR primers) (Genewiz).

### Human embryonic kidney 293T cells

Human embryonic kidney (HEK) 293T cells were cultured in Dulbecco’s modified Eagle’s medium (DMEM) GlutaMAX supplemented with 10% fetal bovine serum (FBS) at 37°C with 5% CO_2_. Cells were transiently transfected directly “in plate” with 1 pg of GPCR plasmid per cell unless otherwise specified. The total DNA amount was maintained at 2 pg per cell with empty vector pcDNA3.1(+). Briefly, the appropriate amount of plasmid DNA was diluted with FluoroBrite DMEM (Live Cell Fluorescence Imaging Medium, without phenol red). In a separate mixture, 2.5 μl of Lipofectamine 2000 per microgram of DNA was diluted in FluoroBrite DMEM and incubated for 5 min before being combined with the DNA mixture and incubated for 20 min. Concurrently, cells were trypsinized, resuspended in 2× supplemented FluoroBrite DMEM (30 mM Hepes, 8 mM glutamine, and 20% FBS), and counted. Cells were mixed with the DNA–Lipofectamine 2000–FluoroBrite DMEM mixture and directly plated onto a black, clear-bottom, tissue culture–treated microplate at the cell density of 5600 cells in 7 μl per well in low volume, LoBase 384-well plates. All microtiter plates were ozone-treated and coated with 0.01% poly-d-lysine.

### Expi293F cells

Expi293F cells, a suspension-adapted cell line derived from HEK293T cells, were cultured and transfected according to the manufacturer’s instructions. Briefly, cells were cultured in serum-free Expi293 medium using culture flasks under constant shaking at 130 rpm at 37°C with 8% CO_2_. For transfection, cells were counted using a Nexcelom Cellometer Auto T4, diluted to 2,000,000 cells/ml, and allowed to grow overnight. The next day, the cells were counted and diluted to 3,000,000 cells/ml, and 1.25 ml of cells was transferred to each well of a 12-well culture plate. Transient transfections were then performed with the Expifectamine 293 transfection kit (Thermo Fisher Scientific) per the manufacturer’s instructions. Each well of cells was transfected with 4 μl of ExpiFectamine reagent and 0.25 μg of GPCR plasmid DNA. Total transfected plasmid DNA was kept constant at 1.5 μg per well with empty vector pcDNA3.1(+). Enhancers were added 18 to 24 hours after transfection, and cells were harvested 72 hours after transfection. Expi293F cells harvested without transfection were grown for 48 hours in 12-well plates (Sarstedt) with an initial density of 3 × 10^6^ cells/ml in 1.25 ml of volume.

### SK-N-MC cells

SK-N-MC cells [American Type Culture Collection (ATCC) HTB-10, neuroepithelioma cell line] were cultured at 37°C and 5% CO_2_. SK-N-MCs grown for the SBA assay were maintained in DMEM with GlutaMAX and supplemented with 10% FBS, non-essential amino acids, Hepes, penicillin-streptomycin, and sodium pyruvate. Cells were grown in 10-cm round tissue culture dishes (Sarstedt) and harvested at approximately 80% confluency. SK-N-MCs grown for the MolBoolean assay were maintained in Eagle's Minimum Essential Medium (EMEM) supplemented with 10% FBS, cultured at 37°C and 5% CO_2_ in a humidified atmosphere until 80 to 90% confluency, then seeded at a density of 120,000 cells in 0.4 ml per chamber in eight-well chamber slides (Nunc Lab-Tek), and allowed to grow for 24 hours before assay processing.

### SH-SY5Y cells

SH-SY5Y (ATCC CRL-2266, neuroblastoma cell line) cells were cultured at 37°C and 5% CO_2_. The cells were maintained in 10% FBS DMEM with GlutaMAX and supplemented with non-essential amino acids, Hepes, penicillin-streptomycin, and sodium pyruvate. Cells were grown in 10-cm round tissue culture dishes (Sarstedt) and harvested at ~80% confluency.

### IP1 accumulation assay

HEK293T cells were transfected with 1 pg per cell of CALCRL DNA, as described above. Twenty-four hours after transfection, IP1 accumulation assay was performed as previously described (*37*). After incubation for 2 hours, HTRF reagents and IP1 calibration standards were added and incubated for 2 hours in the dark at room temperature (RT). Time-resolved fluorescence signals were read on a BioTek Synergy NEO-TRF Hybrid multi-mode reader (BioTek Instruments). Data were collected from three independent experiments performed in technical triplicate.

CALCRL constructs were assayed by cotransfecting HEK293T cells with different tagged versions of CALCRL alone or with each RAMP and with the promiscuous Gqs5 chimera protein at a DNA ratio of 1:1:0.5. Gqs5 is an engineered Gq protein containing the last five amino acid residues of Gs, which allows Gs-coupled GPCRs to signal through Gq downstream signaling pathways (*38*, *39*).

Data reduction, standard calibration, and transformation of HTRF data were performed as previously described (*37*). Normalized IP1 values for CALCRL and RAMP constructs validation assays were calculated relative to the unstimulated mock-transfected cells (set to 0%) and fully stimulated CALCRL (HA-CALCRL-1D4 or FLAG-CALCRL-1D4) coexpressed with FLAG-RAMP3-OLLAS for validation of CALCRL or HA-CALCRL-OLLAS coexpressed with RAMP3 (FLAG-RAMP3-OLLAS or 3xHA-RAMP3-OLLAS) for validation of each RAMP. These data were fitted to a three parameters sigmoidal dose-response function (Prism 9).

### Clarified lysate preparation

Cell membranes were solubilized with *n*-dodecyl-β-d-maltoside (DM) detergent (Anatrace) to form micelles around membrane proteins and maintain membrane protein structure. Expi293F cells (used for the GPCR-RAMP interactome and the native GPCR-RAMP interactome SBA assays) or SK-N-MC and SH-SY5Y cells (used for the native GPCR-RAMP interactome SBA assay) were harvested and washed twice with cold phosphate-buffered saline (PBS). Cells were then incubated in solubilization buffer [50 mM Hepes, 1 mM EDTA, 150 mM NaCl, and 5 mM MgCl_2_ (pH 7.4)] with 1% (w/v) DM and cOmplete mini protease inhibitor (Roche) for 2 hours at 4°C with nutation. Following solubilization, lysates were clarified by centrifugation at 22,000*g* for 20 min at 4°C. Solubilized lysates were transferred to a microcentrifuge tube, and the total protein content was determined by Protein DC assay according to the manufacturer’s specifications. Solubilized lysates were flash-frozen before storage.

### SBA assays

To generate the SBA, anti-GPCR, anti-RAMP, and anti-tag Abs were covalently coupled to color-coded beads (MagPlex, Luminex Corp.), which are magnetic, bar-coded beads defined by a unique combination of infrared and near-red dyes. Each Ab was coupled to a unique bead ID population, as previously described (*11*). Briefly, 1.75 μg of each Ab was diluted in MES buffer [100 mM 2-(*N*-morpholino)ethanesulfonic acid (pH 5.0)] to a final volume of 100 μl. The carboxylated surface of the magnetic beads was activated with *N*-hydroxysuccinimide (Pierce) and 1-ethyl-3-(3-dimethylaminopropyl)-carbodiimide (ProteoChem). After 20 min of activation, the diluted Abs were conjugated onto the activated carboxylated beads using *N*-hydroxysuccinimide–1-ethyl-3-(3-dimethylaminopropyl)carbodiimide chemistry. After 2 hours of incubation in the dark with nutation, unbound Abs were washed away using PBS containing 0.05% Tween 20 (PBST) before adding Blocking Reagent for ELISA (Roche, ref:11112589001) buffer supplemented with ProClin 300 (Sigma-Aldrich) and overnight incubation. The following day, Ab-coupled beads were subsequently grouped and pooled. The coupling efficiency was determined using anti–rabbit-R-phycoerythrin (RPE) and anti–mouse-RPE Abs (Jackson ImmunoResearch), as most of the Abs coupled onto beads were rabbit pAbs or mouse mAbs. The data were collected using a FlexMap three-dimensional (3D) instrument (Luminex Corp., xPONENT Software, build 4.3.309.1).

The SBA assay was carried out in a 384-well format, following the same protocol described previously (*12*), which is consistent with our proof-of-concept study (*9*). Clarified protein lysates were diluted to 2 μg/μl in solubilization buffer (described above) with 0.01% (w/v) DM in a 96-well unskirted polymerase chain reaction plate and diluted 3.6 times again in SBA assay buffer such that 12.5 μl of the lysate (25 μg) was combined with 32.5 μl of assay buffer to a final volume of 45 μl. The solution was then transferred to a 384-well assay plate containing 5 μl of bead array per well using CyBio SELMA (Analytik Jena).

For the SBA assay to detect native GPCR-RAMP interactions, the following modifications were incorporated into the 384-well assay plate workflow to adapt it to a 96-well format: On the day of the assay, “wild-type” cell lysates were diluted to 4 μg/μl, and any transfected cells were diluted to 2 μg/μl in assay buffer, to maximize signal to noise in the readout.

For all protocols, the lysates and beads were incubated overnight for 16 hours at 4°C. Next, the plates were washed five times with 100 μl (96-well plate assay) or six times with 60 μl (384-well plate assay) of PBST using a BioTek EL406 washer. Next, 50 μl of PE-conjugated anti-tag detection Abs diluted in BRE containing 0.1% DM, 0.1% Tween 20, and 10% rabbit immunoglobulin G was added per well, and the plate was incubated for 1 hour at 4°C. The final dilutions used for the detection Abs across all experiments were as follows: PE-conjugated anti-1D4, 1:1000 and PE-conjugated anti-OLLAS, 1:500. PE-conjugated anti-1D4 and PE-conjugated anti-OLLAS were generated using the anti-1D4 and anti-OLLAS Abs available in-house and a PE conjugation kit (Abcam). For the SBA assay to determine native GPCR-RAMP interactions, detection was enabled using PE-conjugated anti-RAMP1, anti-RAMP2, and anti-RAMP3 Abs diluted 1:200 or PE-conjugated anti-OLLAS Ab diluted 1:500. The starting concentration of each PE-conjugated Ab was 0.83 μg/μl.

After the incubation, the beads were washed three times with 100 μl (96-well plate assay) or six times with 60 μl (384-well plate assay) of PBST, and then 100 μl or 60 μl of PBST was added to the beads (96-well and 384-well assays, respectively). The fluorescence associated with each bead was measured using a Luminex FlexMap 3D instrument (Luminex Corp., xPONENT Software, build 4.3.309.1). The data are reported as MFI.

The number of replicates performed for each SBA assay is as follows: For the GPCR-RAMP interactome screen, one technical replicate was performed for one biological replicate for each unique GPCR-containing sample (i.e., GPCR expressed alone or GPCR coexpressed with RAMP1, RAMP2, or RAMP3). Technical duplicates were included for a subset of samples chosen at random. For the native GPCR-RAMP interactome screen, technical duplicates of biological triplicates for each cell type were performed.

### Data analysis of SBA assays based on the DuET library

Data analysis was performed using R version 3.6.0 (*40*) with visualization using ggplot2 (version 3.4.1) (*41*) or base R unless stated otherwise. Expression of transfected RAMP was evaluated by comparing samples with mock transfection with samples from RAMP1-, RAMP2-, or RAMP3-transfected cells. The comparisons were made using one-way analysis of variance (ANOVA) (*aov* function of the stats package, version 3.6.0) followed by Dunnett’s multiple comparisons tests (*DunnettTest* function of the DescTools package, version 0.99.43) separately for capture using anti-HA, anti-RAMP1, anti-RAMP2, and anti-RAMP3 Abs.

To validate the positive control complex CALCRL-RAMP1, a one-way ANOVA followed by Dunnett’s multiple comparisons tests was performed, comparing mock-transfected cells and CALCRL-RAMP1-transfected cells with empty samples (buffer only). CALCRL expression was validated with anti-FLAG capture and PE anti-1D4 detection. RAMP1 expression was validated with anti-HA capture and anti-OLLAS detection. Complex formation was determined by examining data from anti-RAMP1, anti-HA, or anti-OLLAS capture and anti-1D4 detection or anti-CALCRL, anti-FLAG, or anti-1D4 capture and anti-OLLAS detection.

Reproducibility in detecting GPCR-RAMP complexes was tested by comparing anti-OLLAS or anti-HA capture measurements with anti-1D4 detection or anti-1D4 or anti-FLAG capture with anti-OLLAS detection. The samples tested were solubilized membranes of cells expressing GPCR only or coexpressing a GPCR with RAMP2 or RAMP3. GPRC5A was tested with RAMP2, and HCRTR2 was tested with RAMP3 in biological triplicates and technical duplicates. Differences between GPCR alone and GPCR-RAMP samples were evaluated using the Wilcoxon rank-sum test (*wilcox.test* function of the stats package) per GPCR, per capture-detection scheme.

To put measurements from different Abs on a similar scale, raw MFI values were transformed to R.*Z*-scores separately per capture-detection scheme with the formula [*x* − median(*x*)/1.4826 * MAD(*x*)], with *x* being the measurement values from one Ab.

GPCR-RAMP complex detection with the five different epitope-based capture-detection schemes was determined by applying a threshold for complex detection, which was defined by constructing specificity-selectivity plots. We considered RAMP-specific capture with anti-RAMP1, RAMP2, and RAMP3 pAbs an “epitope-based” scheme as it was generalizable to all GPCRs tested. The specificity and selectivity curves were plotted as a function of the threshold and displayed as R.*Z*-score. Sensitivity is defined as the fraction of known interacting GPCRs that were correctly detected as interacting, and specificity is defined as the fraction of known noninteracting GPCRs that were correctly detected as not interacting, using all previously published reports on GPCR-RAMP interactions (*1*). The intersection of the two curves was assigned as the threshold. Detected interactions per capture-detection scheme and GPCR were visualized in a heatmap generated using the ComplexHeatmap package (version 2.2.0) (*42*).

GPCR-RAMP complex detection with protein-based capture-detection schemes was analyzed by individually defining a passing threshold for complex detection for each anti-GPCR Ab. The threshold was 6 MADs (constant of 1.4826) of the expected negative population for each anti-GPCR Ab added to the population density peak (*12*, *43*). Results were visualized in a heatmap using the ComplexHeatmap package (*42*).

To put epitope-based capture and protein-based capture data on the same scale for comparisons, GPCR-RAMP interactions were classified by the fraction of epitope-based capture schemes or protein-based capture schemes (capture with anti-GPCR Abs) that detected an interaction. An interaction was classified as strong if more than two-third of the capture-detection schemes passed, medium if the fraction of passing capture-detection schemes was between one-third and two-third, and weak if less than one-third of the capture-detection schemes passed.

To investigate whether there were any biases in the detected GPCR-RAMP interactions arising from differences in GPCR or RAMP expression, the proportions of interaction evidence classes (strong, medium, and weak) were visualized across quartiles of GPCR or RAMP expression in bar plots. The visualization was also made for epitope capture schemes separately for proportions of passing or failing interactions.

The amount of strong, medium, or weak GPCR interactions pairs was visualized in UpSet plots (ComplexUpset package, version 1.3.3) (*44*, *45*), separately for epitope-based and protein-based capture (*42*).

### Data analysis of native GPCR-RAMP interactomes

The MFI data were transformed to SNRs by dividing signals per capture and detection Ab by the median of the corresponding buffer signals. Native interactions were evaluated per cell type and detection Ab. SNRs were normalized using quantile normalization (*normalizeBetweenArrays* function of the limma R package, version 3.42.2) to account for potential differences in protein concentration between replicates and then transformed to R.*Z*-scores. Any data points above 3.5 (*46*) were annotated as interactions.

### MolBoolean assay protocol

SK-N-MC cells seeded onto chamber slides were fixed with 3.7% paraformaldehyde (PFA) and permeabilized with 0.2% (v/v) Triton X-100 after 24 hours, as described in (*20*). After fixation, cells were washed twice in tris-buffered saline and then processed following the instructions for the MolBoolean assay kit provided by Atlas Antibodies (protype product based on (*20*), gift from Atlas Antibodies). Although the original MolBoolean assay was designed for mouse and rabbit primary Abs, we used a MolBoolean kit developed by Atlas Antibodies for use with sheep and rabbit primary Abs. The Abs for staining endogenous GPCRs and RAMPs (table S1) were used at a final concentration of 0.002 mg/ml for GPCR Abs and 0.015 mg/ml for RAMP Abs. Samples were incubated with primary Abs overnight at 4°C. After MolBoolean processing, cells were mounted in DuoLink mounting medium with 4′,6-diamidino-2-phenylindole (DAPI; Sigma-Aldrich), incubated at RT, stored overnight at 4°C, and imaged the following day.

### MolBoolean image acquisition and processing

Deconvoluted images were acquired with a DeltaVision Image Restoration Inverted Olympus IX-71 microscope on the blue, red, and far-red channels using a 60× oil immersion objective. Excitation/emission wavelengths are 390 ± 18/435 ± 48 nm for the blue channel (DAPI), 575 ± 25/632 ± 60 nm for the red channel, and 632 ± 22/676 ± 34 for the far-red channel. Exposure times and transmittance percentages were constant while imaging all samples in the same experiment. At least three Z-stack images (0.2-μm thickness per slice) of different fields of view were captured per coverslip for each sample.

Image processing was done in ImageJ (adding scale bars, generating maximum projections, and generating split channel images) and CellProfiler (*47*). Quantification of RCPs per channel and quantification of overlapping RCPs were carried out in CellProfiler with a pipeline developed by Raykova *et al.* and provided by Atlas Antibodies (*20*, *47*). The same pipeline parameters were used for all samples within an experiment (threshold = 0.5 for the RAMP staining and 0.25 to 1.0 for the GPCR staining). Nuclei were quantified manually. Adjustments for brightness and contrast in ImageJ were made on images for visualization purposes only.

### MolBoolean data analysis

Total RCPs for each Z-stack were divided by the total number of cells per image, and the number of RCPs from the “blank” sample (cells that were not stained with any primary Ab) was subtracted to obtain net RCPs per cell (Excel). To calculate net normalized RCPs per cell, the number of RCPs corresponding to complexed and uncomplexed proteins was normalized to the total number of RCPs per cell. The net RCPs per cell and net normalized RCPs per cell data were plotted in Prism 9 (GraphPad). Statistical significance was determined by a one-way ANOVA followed by Dunnett’s multiple comparisons test to the positive control using R version 3.6.0 (*aov* function of the stats package, version 3.6.0 and *DunnettTest* function of the DescTools package, version 0.99.43) (*40*).

### Bioinformatic analysis and GPCR-RAMP interaction annotation

GPCRs were annotated as RAMP-interacting or not based on the SBA assay screen results (fig. S11A). GPCRs with strong evidence for complex formation with a particular RAMP for both epitope-based and protein-based capture schemes or with strong evidence for complex formation as determined by one capture scheme and medium evidence as determined by the other were assigned as interacting (yes). The GPCRs with weak evidence for interaction with a particular RAMP for both capture schemes and the GPCRs with weak evidence for interaction from one scheme and medium evidence for interaction from the other were assigned as noninteracting (no). GPCRs without definitive evidence for interaction or noninteraction for each RAMP were classified as inconclusive and excluded from subsequent analysis. The annotation for each GPCR was RAMP specific. A GPCR lacking protein capture data was assigned yes if it demonstrated strong evidence for interaction with a given RAMP by epitope-based capture only and no if it exhibited weak evidence for interaction with a given RAMP by epitope-based capture only.

### Gene expression analysis

The RNA consensus tissue gene data and RNA single-cell–type data files were downloaded from the HPA webpage (proteinatlas.org) (*21*, *22*). The tissue dataset contains consensus transcript expression levels summarized for each gene in 50 tissues based on transcriptomic data from the HPA and Genotype-Tissue Expression (GTEx) (The HPA version 23.0 and Ensembl version 109). The single-cell dataset contains transcript expression levels summarized for each gene in 81 cell types from 31 datasets. The nTPM expression values were used in the analysis for both datasets.

The expression ratio between the GPCR and each RAMP for each cell type was calculated if the nTPM was greater than one for both genes. Violin plots were made using the R package ggplot2 (v. 3.4.0). Significances between expected interactors and noninteractors were calculated with two-sided Wilcoxon test using *wilcox.test* function from stats package (v. 4.2.1).

To explore the RAMP expression across the different human tissues and cell types, we first set a binary cutoff where expressions greater than 1 nTPM were considered expressed, and values less than 1 nTPM were not considered expressed. A heatmap was generated to visualize the differences in expression levels for the different RAMPs in human cells. Cells with nTPM values less than 1 were assigned a 0 value, and the cells with expression above 1 nTPM were log_2_-transformed. The heatmap was produced using R package pheatmap (v. 1.0.12). Hierarchical clustering analysis was performed using *hclust* function from stats R package (v. 4.2.1). The percentage of interacting and noninteracting GPCRs per cell type and per RAMP was calculated for the cell types where GPCR and RAMP expression was greater than 1 nTPM.

### Data analysis of G protein and βArr couplings

G protein and βArr couplings were compared between groups of GPCRs that were found to interact with any RAMP and GPCRs that did not interact. Coupling data were downloaded from the GPCRdb (https://gpcrdb.org/) for all GPCRs in the study with information available (*23*–*26*). βArr couplings were binarized on the basis of the log(*E*_max_/*E*50) values, where 0 was treated as no coupling and >0 as coupling. The kinds of G proteins annotated as being primary or secondary couplers for a specific GPCR, the total number of primary G protein couplings, and the kinds of βArr couplings for each GPCR were compared between RAMP-interacting and noninteracting GPCRs using the Fisher exact test (*fisher.test* R function).

### Interactive web interface

A web-based companion R app was made using the shiny package (version 1.7.1) and R version 4.2.0. The Shiny app was created to contain information on interactions for individual GPCRs, which were visualized in heatmaps (ComplexHeatmap package, 2.14.0), circle plots (circlize package, 0.4.15) (*48*), and density plots (ggplot2 package, 3.3.6) (*41*). Summaries of interactions were visualized in heatmaps (plotly package, 4.10.0) (*49*). The interface was containerized with Docker (version 25.0.2, build 29cf629) and hosted online on the shinyapps.io platform.
