# Supplementary material for: Multiplexed mapping of the interactome of GPCRs with receptor activity–modifying proteins
Source: Sci Adv. 2024 Jul 31;10(31):eado9959. doi: 10.1126/sciadv.ado9959 (PMC11290489; doi:10.1126/sciadv.ado9959)
Supplement: Supplementary file 1 — Figs. S1 to S13 Tables S2, S4, S5, and S8 Legends for tables S1, S3, S6, and S7 [file sciadv.ado9959_sm.pdf]

Science Advances
AAAS

Supplementary Materials for

Multiplexed mapping of the interactome of GPCRs with receptor activity–modifying proteins

Ilana B. Kotliar *et al.*

Corresponding author: Jochen M. Schwenk, jochen.schwenk@scilifelab.se;
Thomas P. Sakmar, sakmar@rockefeller.edu

Sci. Adv. **10**, eado9959 (2024)
DOI: 10.1126/sciadv.ado9959

The PDF file includes:

Figs. S1 to S13
Tables S2, S4, S5, and S8
Legends for tables S1, S3, S6, and S7

Other Supplementary Material for this manuscript includes the following:

Tables S1, S3, S6, and S7

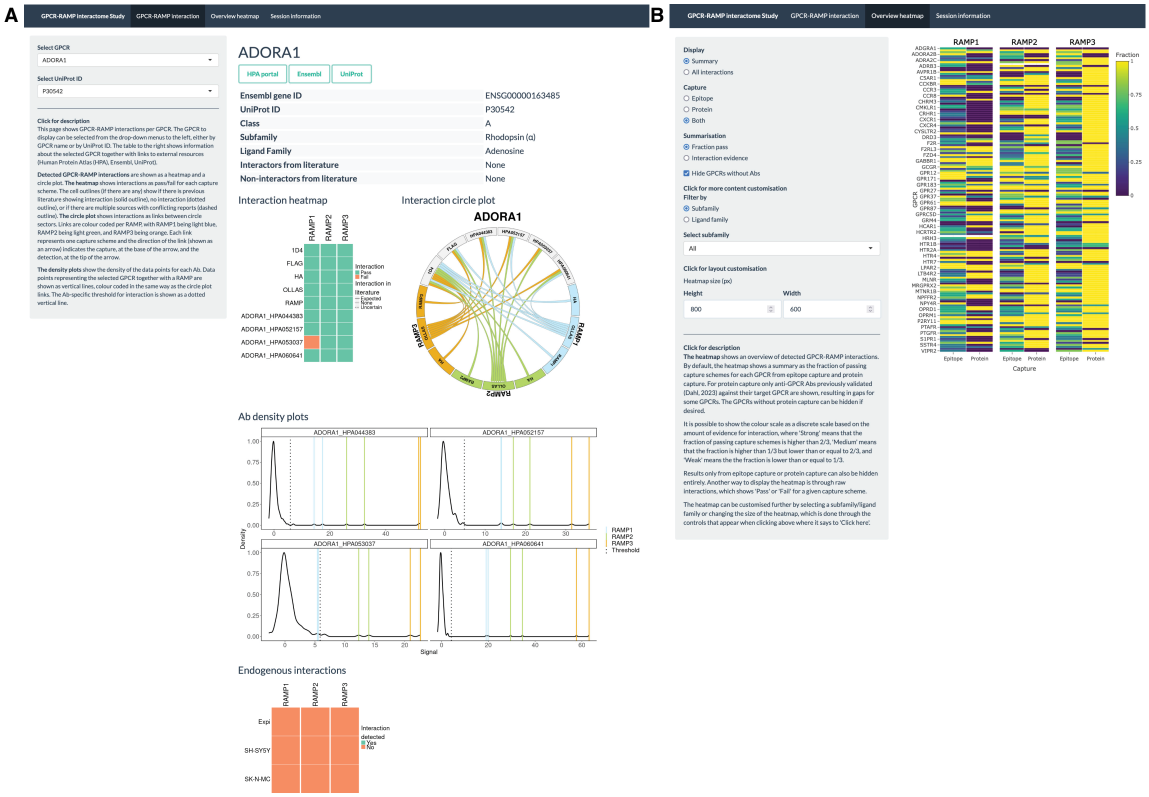

Fig. S1.

Web interface to browse the GPCR-RAMP interactome.

The results from the GPCR-RAMP interactome analysis are available in an R-based Shiny app that can be accessed in a web browser. The web interface presents a summary of GPCR-RAMP interactions detected for all possible capture-detection schemes. (**A**) The display for the adenosine A1 receptor (ADORA1) is presented as an example. Results are displayed as a binary heatmap and alluvial plot, and the density plots for the GPCR-specific capture of GPCR-RAMP complexes with anti-GPCR Human Protein Atlas (HPA) Abs are shown. A similar display is available on the app for each GPCR tested. (**B**) The app allows the user to customize the analysis of the GPCR-RAMP interactome dataset to create heatmap displays.

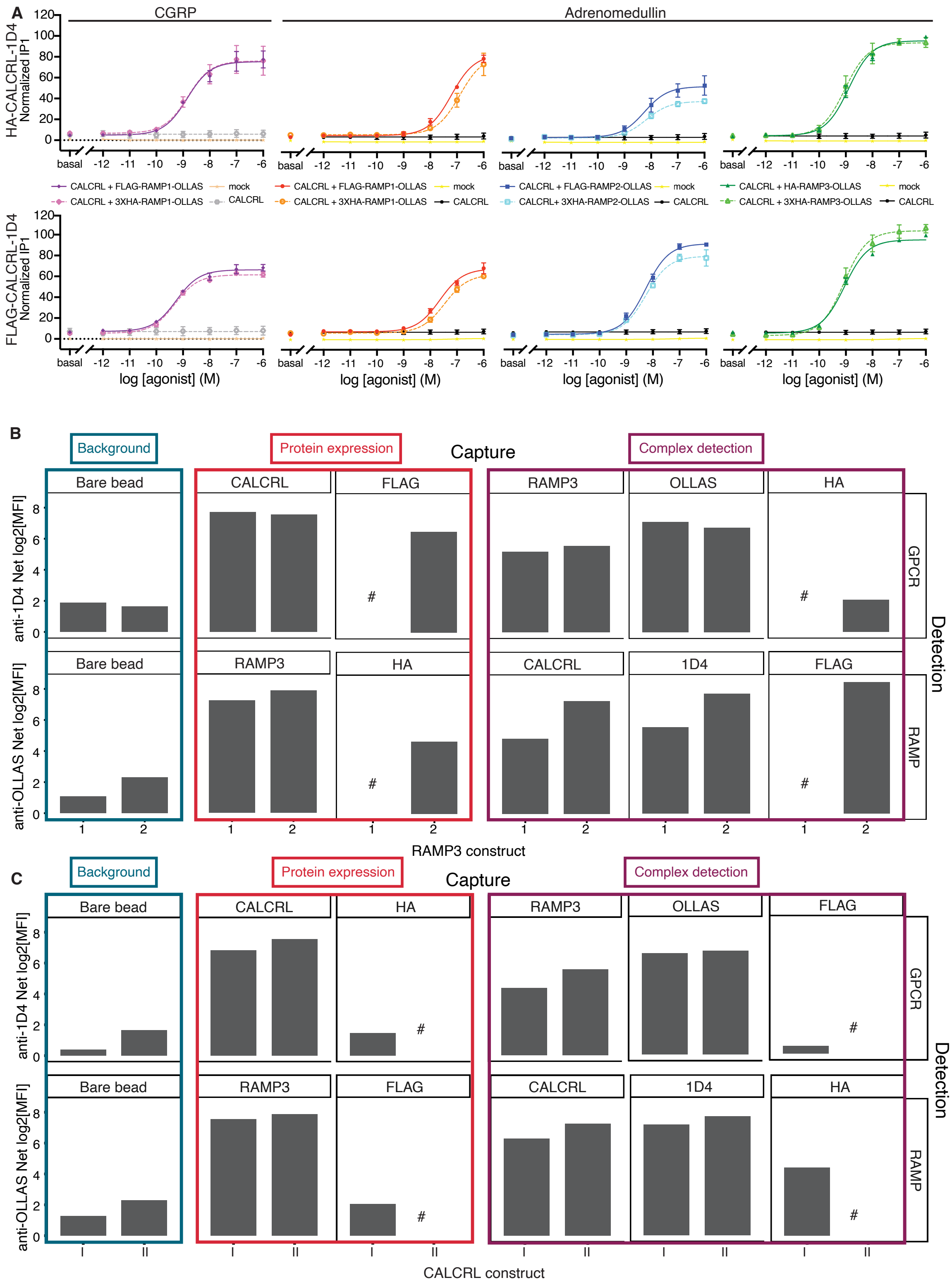

A
B
C
CGRP
Adrenomedullin
HA-CALCRL-1D4 Normalized IP1
FLAG-CALCRL-1D4 Normalized IP1
log [agonist] (M)
CALCRL + FLAG-RAMP1-OLLAS
CALCRL + 3XHA-RAMP1-OLLAS
CALCRL + FLAG-RAMP2-OLLAS
CALCRL+ 3XHA-RAMP2-OLLAS
CALCRL + HA-RAMP3-OLLAS
CALCRL + 3XHA-RAMP3-OLLAS
mock
CALCRL
Background
Protein expression
Complex detection
Capture
Detection
Bare bead
CALCRL
FLAG
RAMP3
OLLAS
HA
1D4
GPCR
RAMP
anti-1D4 Net log2[MFI]
anti-OLLAS Net log2[MFI]
RAMP3 construct
CALCRL construct

Fig. S2.

Validation of GPCR-RAMP complexes used as positive controls.
(**A**) IP1 accumulation assays were carried out in HEK293T cells expressing each RAMP construct used in this study (3xHA-RAMP-OLLAS) or each of the previously validated RAMP constructs (FLAG-RAMP-OLLAS) (*9*) in the presence of dual epitope-tagged CALCRL (top row, HA-CALCRL-1D4 or bottom row, FLAG-CALCRL-1D4). Dose-response curves were determined for the specified CALCRL with adrenomedullin (all RAMPs) or calcitonin gene-related peptide (CGRP) (RAMP1 only). IP1 accumulation is normalized to maximal adrenomedullin stimulated- HA-CALCRL-1D4 + RAMP3 (top row) or FLAG-CALCRL-1D4 + RAMP3 (bottom row). Data are expressed as the mean ± SEM of normalized IP1 accumulation from three independent experiments performed in four technical replicates. Fitting parameters are provided in **Table S2**. (**B, C**) The constructs described in panel A were used in SBA assays. (**B**) Expi293F cells were co-transfected with FLAG-CALCRL-1D4 in addition to either FLAG-RAMP3-OLLAS (Construct 1) (*9*) or 3xHA-RAMP3-OLLAS (Construct 2). (**C**) Expi293F cells were co-transfected with 3xHA-RAMP3-OLLAS in addition to either HA-CALCRL-1D4 (Construct I) (*9*) or FLAG-CALCRL-1D4 (Construct II). Cells were then solubilized and incubated with the SBA, which included beads conjugated to each of four mAbs targeting HA, 1D4, FLAG, and OLLAS and two previously validated pAbs targeting CALCRL and RAMP3 (*9*). CALCRL-RAMP3 complexes were captured onto the beads, and either CALCRL, RAMP3 or the CALCRL-RAMP3 complex was detected using PE-conjugated anti-1D4 mAb (top row) or anti-OLLAS mAb (bottom row). *Dark turquoise box*: background level (bare bead capture: 1D4, and OLLAS detection). *Red box*: protein expression (CALCRL expression: CALCRL capture, 1D4 detection and FLAG capture, 1D4 detection. RAMP3 expression: RAMP3 capture, OLLAS detection, and HA capture, OLLAS detection). *Plum box*: CALCRL-RAMP3 complex detection (RAMP3 capture, 1D4 detection; OLLAS capture, 1D4 detection; HA capture, 1D4 detection; CALCRL capture, OLLAS detection; 1D4 capture, OLLAS detection; and FLAG capture, OLLAS detection). # indicates that the capture-detection scheme data are omitted for clarity since the tag being captured (FLAG or HA) is present on either CALCRL and RAMP3, or neither. The y-axis represents the 2-fold change in median fluorescence intensity (MFI) compared to samples with mock-transfected lysate.

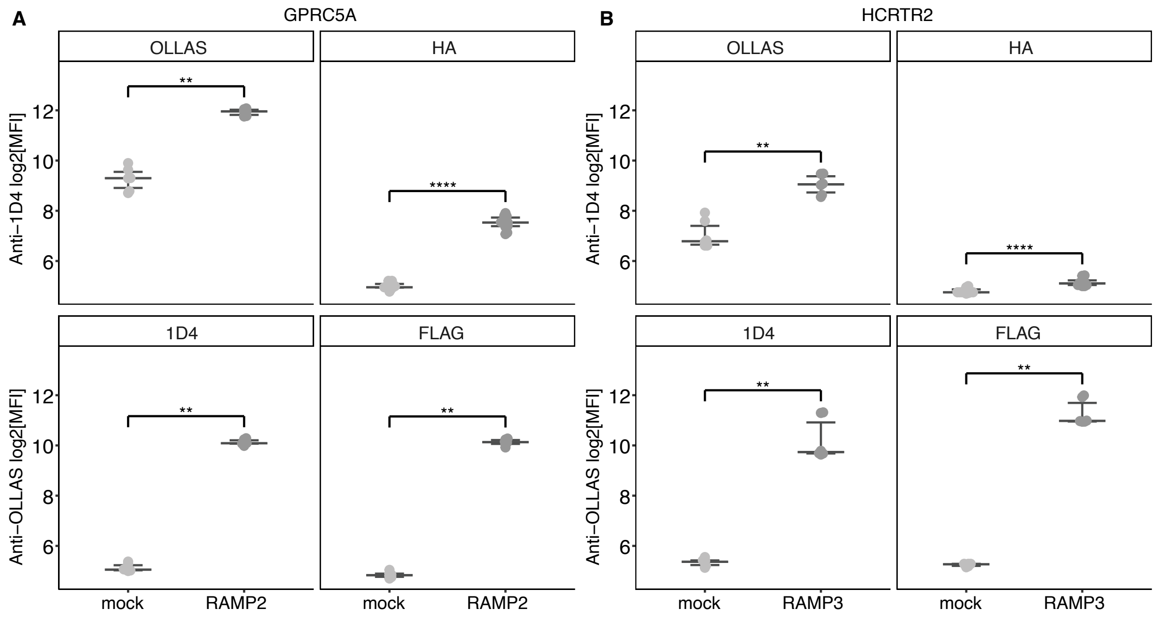

Fig. S3.

Reproducibility of the SBA method to detect GPCR-RAMP complexes.
The reproducibility of the SBA assay for detecting solubilized GPCR-RAMP complexes was tested. Representative examples of GPCRs were selected from two subfamilies. The GPCRs were expressed with or without a RAMP in biological triplicate, and each sample was quantified in technical duplicate (N=6). GPCR class C group 5 member A (GPRC5A; glutamate subfamily) was expressed with or without RAMP2 (**A**), and orexin receptor type 2 (HCRTR2; beta subfamily) was expressed with or without RAMP3 (**B**). Each complex was detected using four epitope-based capture-detection strategies in parallel. *Top row*: the RAMP was captured with beads coupled to anti-HA or anti-OLLAS mAb, and the GPCR was detected with PE-conjugated anti-1D4 mAb. *Bottom row*: the GPCR was captured with beads coupled to anti-1D4 or anti-FLAG mAb, and the RAMP was detected with PE-conjugated anti-OLLAS mAb. Significance was determined by a one-sided unpaired Wilcoxon test (**** $p < 0.0001$, ** $p < 0.01$). Sample sizes and p-values are listed in **Table S3**. X-axis labeling: mock indicates that only the GPCR was ectopically expressed; RAMP2 (**A**) or RAMP3 (**B**) indicate that the GPCR was co-expressed with the specified RAMP. Data are plotted as the log2 of median fluorescence intensity (MFI), and horizontal bars represent the medians of each group with the 25th and 75th percentiles.

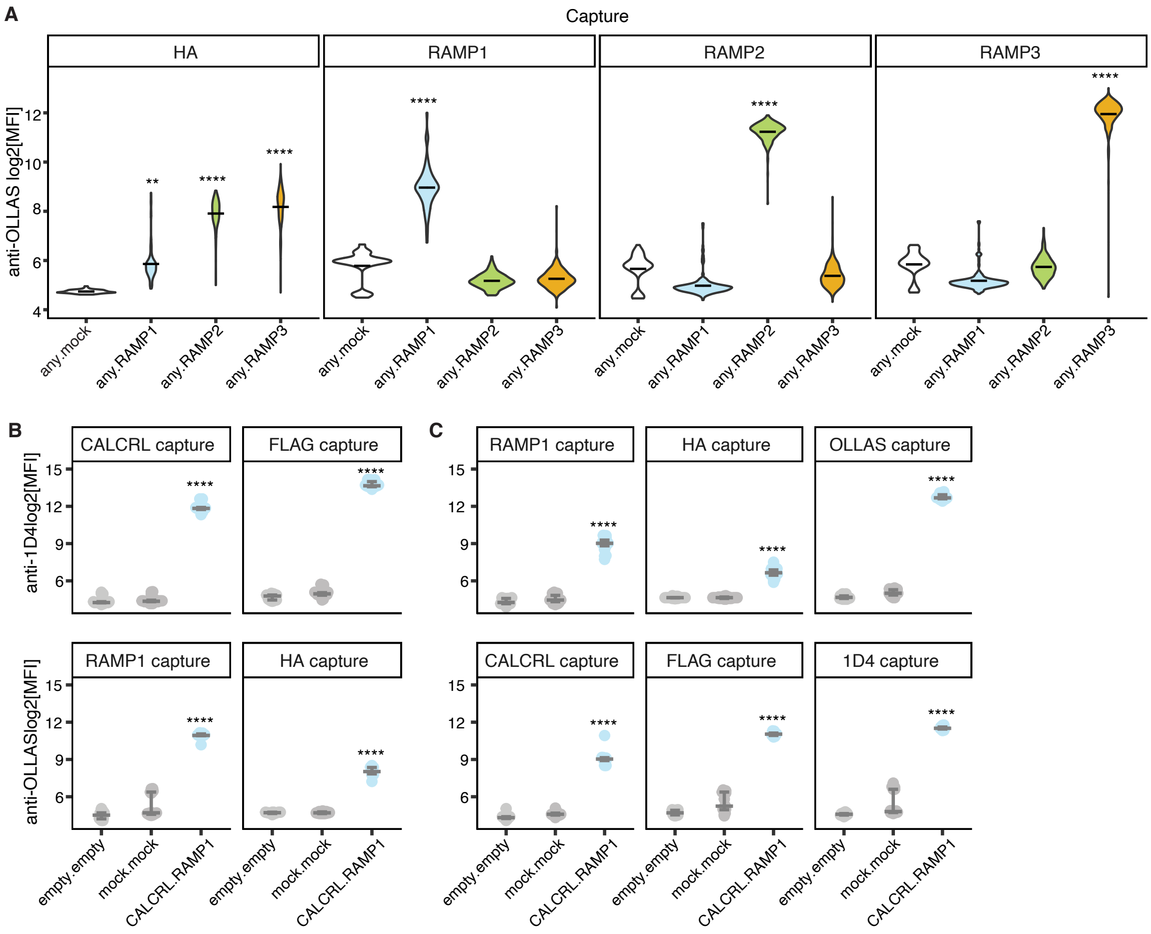

Fig. S4.

RAMP expression and CALCRL-RAMP1 complex formation.
(**A**) Quantification of expressed RAMPs. Relative quantities of solubilized dual epitope-tagged RAMP 1, 2 and 3 were determined by SBA assay. Each RAMP was captured with beads coupled to anti-HA mAb or anti-RAMP specific pAb capture target as noted at the top of each plot and detected with PE-conjugated anti-OLLAS mAb. Violin plot horizontal lines indicate the mean. Significance was determined by a one-way ANOVA (with $p < 0.05$) followed by Dunnett's multiple comparison test to any.mock (**** $p < 0.0001$, ** $p < 0.01$, if not marked $p >= 0.05$). White, any.mock; light blue, any.RAMP1; lime, any.RAMP2; orange, any.RAMP3. (**B, C**) Validation of expression (**B**) and complex detection (**C**) of the positive control used for the SBA assay screen, CALCRL-RAMP1. Lysates from Expi293F cells co-transfected with epitope-tagged CALCRL and epitope-tagged RAMP1 were incubated with the SBA and CALCRL-RAMP1 complexes were captured on the beads in a multiplexed fashion. The proteins were detected by PE-conjugated anti-1D4 mAb (top row) or PE-conjugated anti-OLLAS mAb (bottom row). (**B**) CALCRL expression (top row) and RAMP1 expression (bottom row) were measured with the capture-detection schemes shown. (**C**) CALCRL-RAMP1 complex formation was measured with six capture-detection schemes: RAMP1 was captured with beads coupled to anti-RAMP1 pAb, anti-HA mAb or anti-OLLAS mAb, and the CALCRL-

RAMP1 complex was detected with PE-conjugated anti-1D4 mAb. CALCRL was captured with beads coupled to anti-CALCRL pAb, anti-FLAG mAb or anti-1D4 mAb, and the CALCRL-RAMP1 complex was detected with PE-conjugated anti-OLLAS mAb. Statistical significance was determined by ordinary one-way ANOVA followed by Dunnett's multiple comparisons test to mock.mock (**** $p < 0.0001$, if not marked $p >= 0.05$). The thick horizontal lines represent the median values, and the thin lines above and below show the 75th and 25th percentiles, respectively. Sample names on the x-axis use the format "transfected GPCR name (if any).transfected RAMP name (if any)". Data are plotted as the log2 of median fluorescence intensity (MFI). Sample sizes and p-values are listed in **Table S3**.

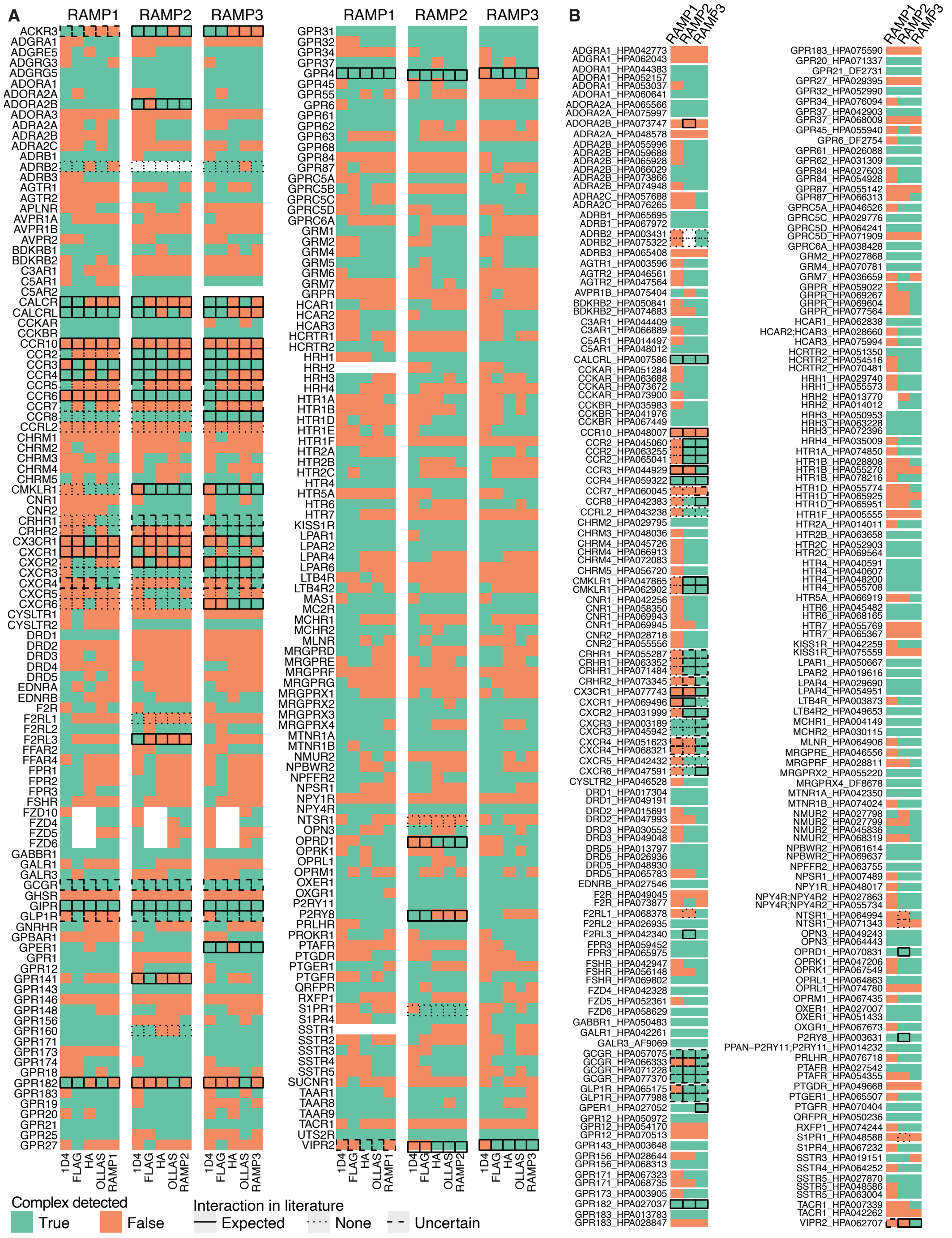

Fig. S5.

Summary of GPCR-RAMP interactome data.
Heatmaps are shown for GPCR-RAMP interactions identified for each RAMP using multiple capture-detection schemes. (**A**) Epitope-based capture. Each row represents a single GPCR. (**B**) Protein-based capture. Each row represents a single anti-GPCR Ab labeled with the format “GPCR target_Ab code.” Different Abs targeting the same GPCR are grouped. Information regarding whether specific GPCR-RAMP interactions are

reported in the literature is overlaid on respective cells as a solid black outline (interaction expected), a thin dashed outline (interaction not expected) or a thick dashed outline (interaction uncertain due to conflicting reports). White boxes indicate GPCR-RAMP pairs not tested due to preparation error. *Green squares*, complex detected; *orange squares*, complex not detected.

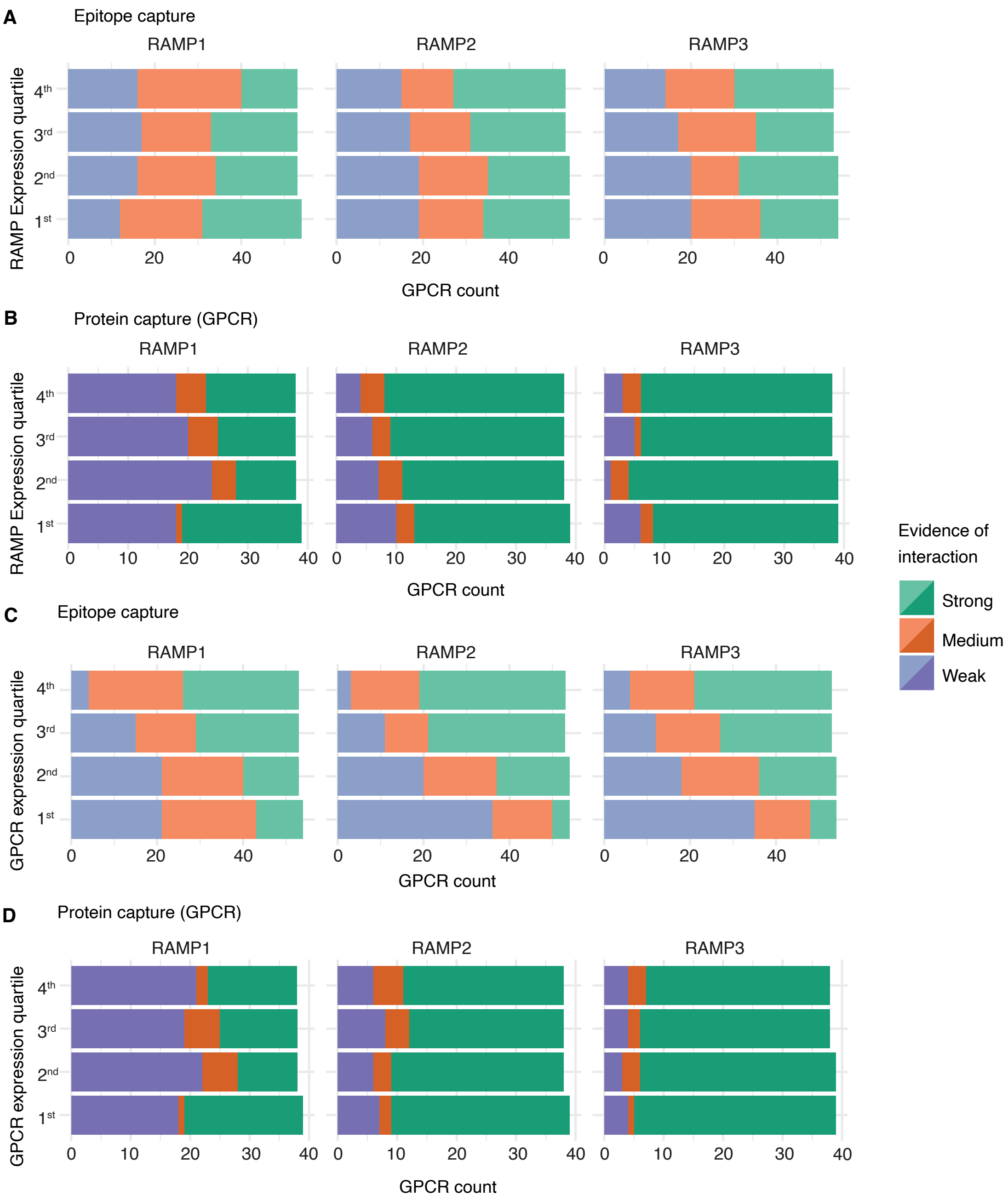

Fig. S6.

Analysis of expression level bias for detecting GPCR-RAMP interactions.
Stacked horizontal bar plots demonstrate the distribution of GPCR-RAMP interactions by evidence class (*green*, strong; *orange*, medium; *purple*, weak). Strong: >66% passing capture-detection schemes. Medium: 33-66% passing capture-detection schemes. Weak: <33% passing capture-detection schemes. The plots are segmented based on RAMP expression levels (**A, B**) or GPCR expression levels (**C, D**). GPCR-RAMP complexes were identified with epitope-based capture of the GPCR and RAMP (**A, C**) or protein-specific capture of the GPCR (**B, D**). There were five unique epitope-based capture-detection schemes and up to six unique protein-based capture-detection schemes for each

GPCR-RAMP pair studied. Protein capture data were generated only for the 154 GPCRs (out of 215) with at least one validated anti-GPCR Ab available. The lowest GPCR or RAMP expression levels correspond to the first quartile and highest correspond to the fourth quartile.

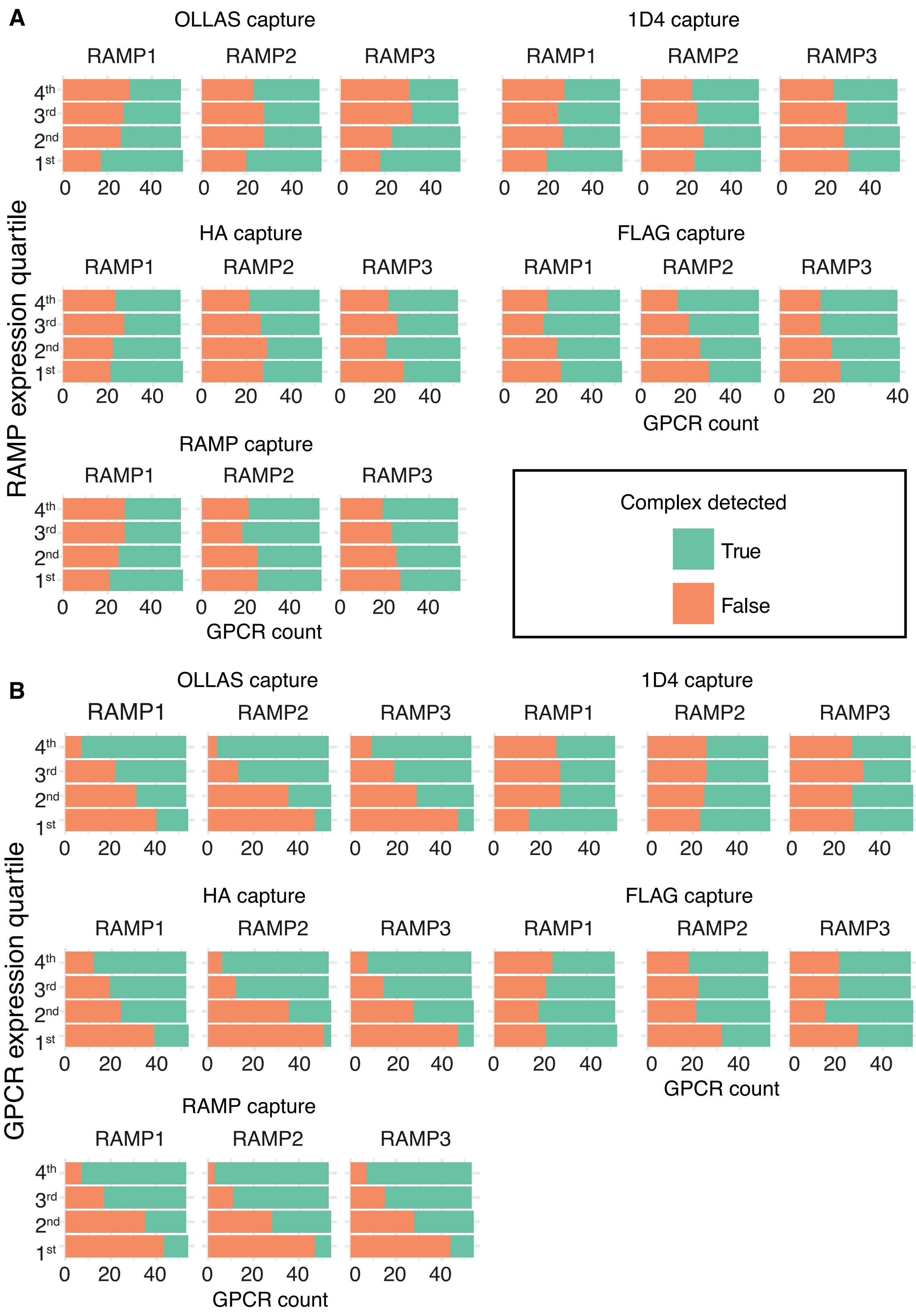
A
OLLAS capture
1D4 capture
RAMP1
RAMP2
RAMP3
HA capture
FLAG capture
RAMP capture
RAMP expression quartile
4th
3rd
2nd
1st
0
20
40
GPCR count
Complex detected
True
False
B
GPCR expression quartile

Fig. S7.

Analysis of expression level bias for epitope-based detection.
Stacked horizontal bar plots demonstrate the distribution of the total number of GPCR-RAMP complexes detected (*green*) or not detected (*orange*) for each RAMP with five different epitope-based capture detection schemes. The plots are segmented based on RAMP expression levels (**A**) or GPCR expression levels (**B**). *Left column*, the three capture-detection schemes are used to capture the RAMP and detect the GPCR. *Right column*, the two schemes used to capture the GPCR and detect the RAMP. The lowest GPCR or RAMP expression levels correspond to the first quartile and highest correspond to the fourth quartile.

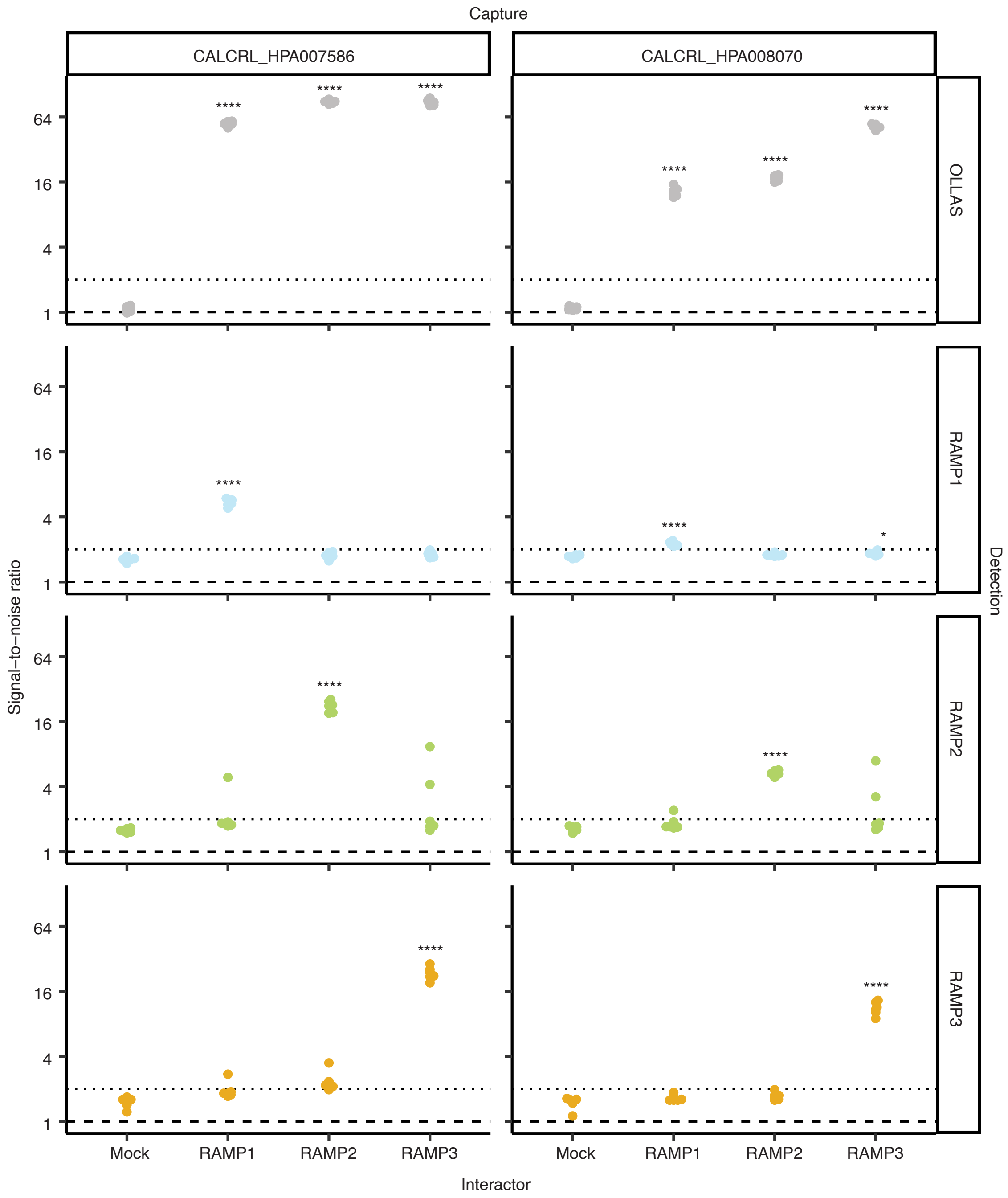

Fig. S8.

Validation of PE-conjugated anti-RAMP Abs for detecting CALCRL-RAMP complexes.
Validation of the detection of CALCRL co-expressed pairwise with RAMP1/2/3. Samples of solubilized membranes from Expi293F cells transfected with epitope-tagged CALCRL alone or co-transfected with each epitope-tagged RAMP were incubated with the SBA. CALCRL-RAMP complexes were captured on the beads with two previously

validated anti-CALCRL HPA Abs. The complexes were detected using PE-conjugated anti-OLLAS mAb (top row) or PE-conjugated anti-RAMP1, anti-RAMP2 or anti-RAMP3 pAb (remaining rows). Statistical significance was determined by ordinary one-way ANOVA followed by Dunnett's multiple comparisons test to mock (*i.e.*, CALCRL expressed alone) (**** $p < 0.0001$, * $p < 0.01$, if not marked $p >= 0.05$). Sample names on the x-axis denote transfected RAMP name (if any). Data plotted as signal-to-noise ratio (unitless) on a log2 scale. Signal-to-noise ratios: dashed line, 1; dotted line, 2. Sample sizes and p-values are listed in **Table S3**.

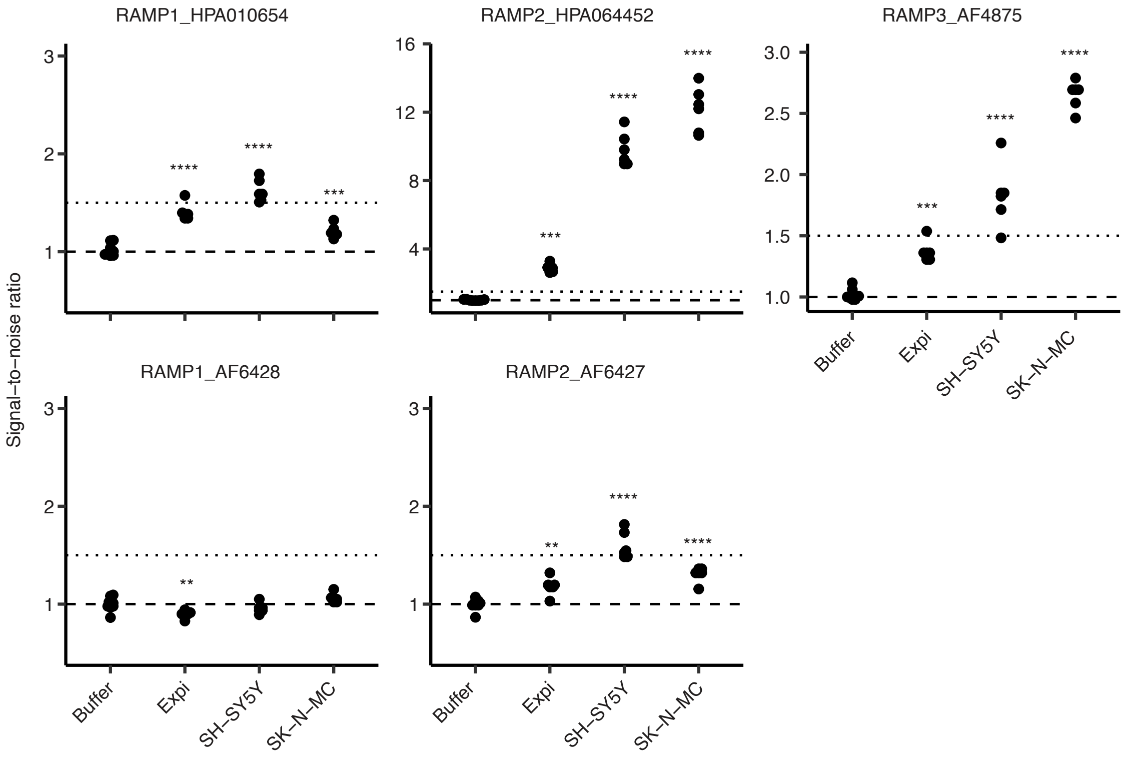

Fig. S9.

Detection of native RAMP expression in three cell lines.
Samples of solubilized lysates from Expi293F (Expi), SH-SY5Y and SK-N-MC cells were incubated with the SBA, which could capture native RAMP1, RAMP2 and RAMP3 with anti-RAMP specific Abs (Ab names are given above each plots). The proteins were detected by PE-conjugated anti-RAMP1, anti-RAMP2 or anti-RAMP3 pAbs. Significance was determined by an ordinary one-way ANOVA with $p < 0.05$ followed by Dunnett's multiple comparison test to buffer (** $p < 0.01$, *** $p < 0.001$, **** $p < 0.0001$, $p >= 0.05$ if not marked). Data plotted as signal-to-noise ratios (unitless). Signal-to-noise ratios: dashed line, 1; dotted line, 1.5. Sample sizes and p-values are listed in **Table S3**.

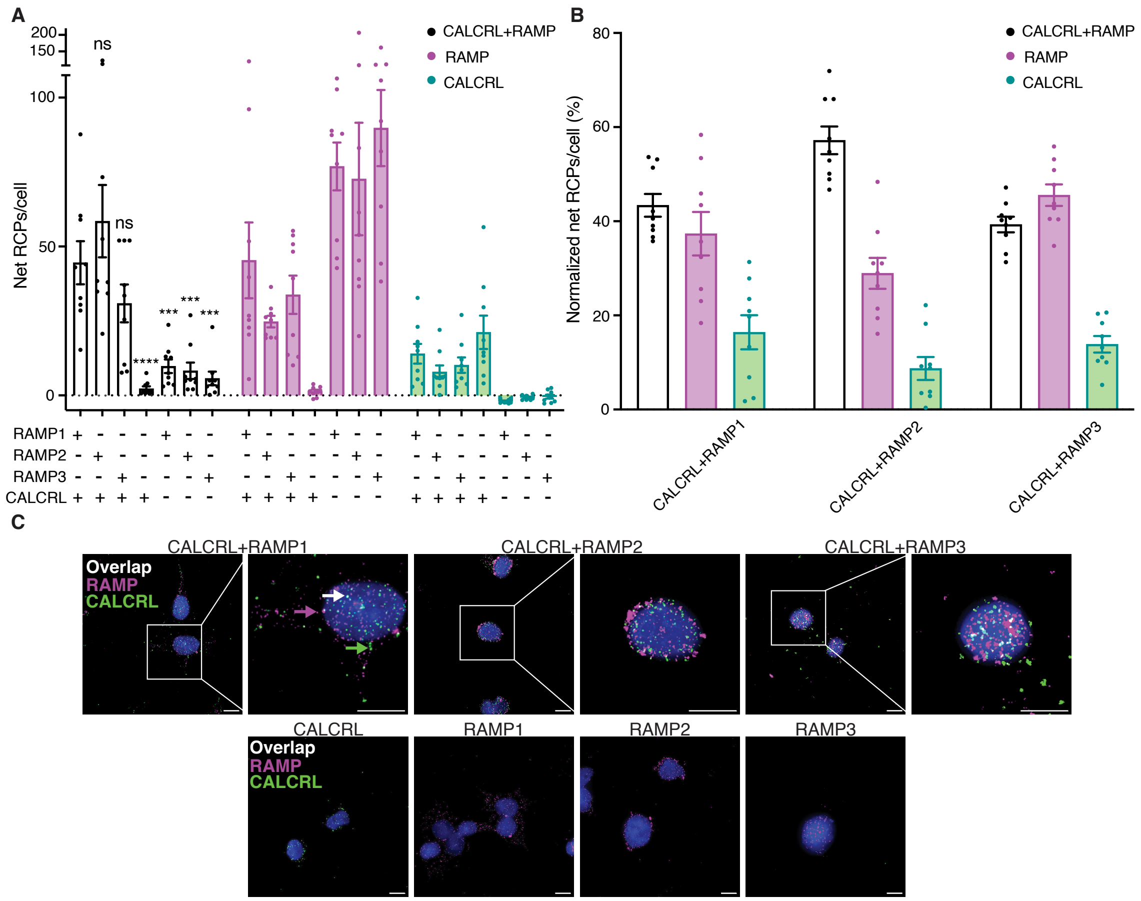

Fig. S10. MolBoolean assay to detect CALCRL-RAMP interactions in cell membranes.

SK-N-MC cells were incubated with anti-CALCRL primary Ab and either anti-RAMP1, anti-RAMP2 or anti-RAMP3 primary Ab and subjected to MolBoolean assay. (**A**) Signals for complexed and un-complexed CALCRL and RAMP were plotted and compared between cells stained with two primary Abs or just one. Results are shown as the number of net rolling circle amplification products (RCPs) per cell. Statistical significance was determined by one-way ANOVA followed by Dunnett's multiple comparisons test to CACLRL-RAMP1 for 'CALCRL+RAMP' (Black bars and dots) only (****$p < 0.0001$; ***$p < 0.001$; ns, not significant). Sample sizes and p-values are listed in **Table S3**. (**B**) Quantification of the percentage of RCPs out of the total number of RCPs/cell (normalized net RCPs/cell) for cells that received anti-CALCR and anti-RAMP primary Abs. (**C**) Representative Z-stack maximum projection images. Scale bars, 10 µm. *Blue*, DAPI; *green*, CALCRL puncta; *magenta*, RAMP puncta; *white*, CALCR-RAMP complex puncta. Colored arrows point to examples of puncta. RCPs from negative control MolBoolean-stained cells not incubated with any primary Ab were subtracted to obtain net RCP values. Labels on the x-axis in (**A**) and (**B**) and on top of the images in (**C**) indicate the target(s) of the included Abs. Data are from three biological replicates performed with at least three technical replicates.

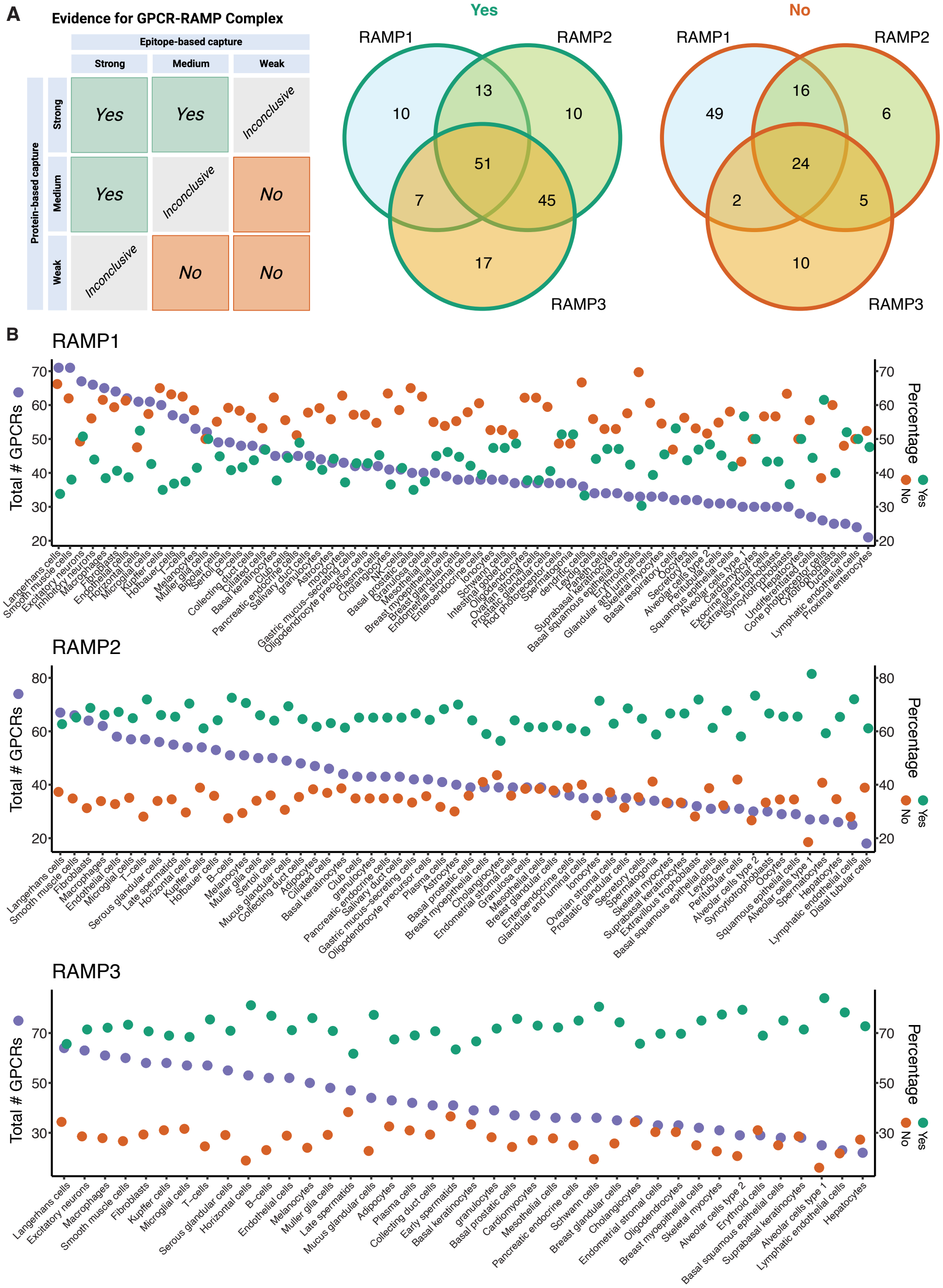

Fig. S11.

Analysis of GPCR expression in human cells parsed by RAMP interaction.

(**A**) *Left*, Matrix showing the assignment of GPCR interaction with a particular RAMP as either yes (*green*), no (*orange*), or inconclusive (*grey*) based on the evidence class of the results from each of the two GPCR-RAMP complex capture schemes: epitope-based or

protein-based capture. A GPCR lacking protein-capture data (not shown in the matrix) was assigned "yes" if it demonstrated strong evidence for interaction with a given RAMP by epitope-based capture only, and "no" if it exhibited weak evidence for interaction with a given RAMP by epitope-based capture only. The resulting dataset included 205 unique GPCRs (172 for RAMP1, 170 for RAMP2, and 161 for RAMP3). In total there were 81 "yes" and 91 "no" GPCRs for RAMP1, 119 "yes" and 51 "no" for RAMP2, and 120 "yes" and 41 "no" for RAMP3. *Middle and right*, Venn diagrams comparing the number of GPCRs that are annotated as "yes" (*green outline*) or "no" (*orange outline*) for each of the three RAMPs to visualize the intersection of the datasets across RAMP subtypes. Generated with Biorender.com (**B**) Scatter plot of the number of expressed GPCRs (*purple dots*) in different human cell types arranged in descending order. Only GPCRs annotated as "yes" or "no" as described in (**A**) for interaction with a specified RAMP were examined. Only GPCRs with nTPM >1 in cells with RAMP expression nTPM>1 are shown (*left axis*). Plots are based on RNASeq single-cell data (proteinatlas.org) (*21, 22*). The percentage of GPCRs annotated as "yes" (*green dot*) or "no" (*orange dot*) for interaction with each RAMP is overlaid for each cell type shown (*right axis*).

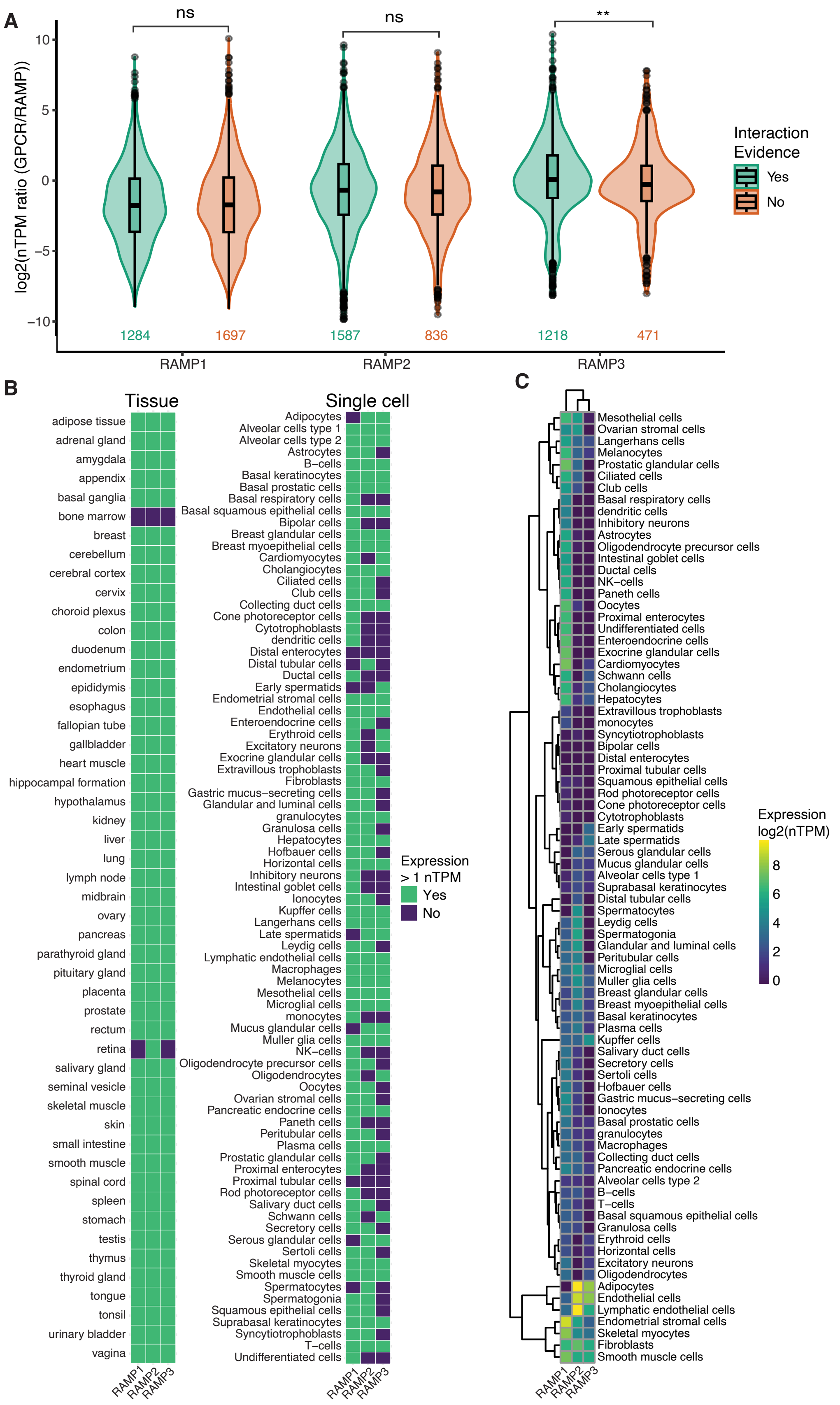

A
ns
ns
**
10
5
0
−5
−10
log2(nTPM ratio (GPCR/RAMP))
1284
1697
1587
836
1218
471
RAMP1
RAMP2
RAMP3
Interaction Evidence
Yes
No
B
Tissue
adipose tissue
adrenal gland
amygdala
appendix
basal ganglia
bone marrow
breast
cerebellum
cerebral cortex
cervix
choroid plexus
colon
duodenum
endometrium
epididymis
esophagus
fallopian tube
gallbladder
heart muscle
hippocampal formation
hypothalamus
kidney
liver
lung
lymph node
midbrain
ovary
pancreas
parathyroid gland
pituitary gland
placenta
prostate
rectum
retina
salivary gland
seminal vesicle
skeletal muscle
skin
small intestine
smooth muscle
spinal cord
spleen
stomach
testis
thymus
thyroid gland
tongue
tonsil
urinary bladder
vagina
RAMP1
RAMP2
RAMP3
Single cell
Adipocytes
Alveolar cells type 1
Alveolar cells type 2
Astrocytes
B−cells
Basal keratinocytes
Basal prostatic cells
Basal respiratory cells
Basal squamous epithelial cells
Bipolar cells
Breast glandular cells
Breast myoepithelial cells
Cardiomyocytes
Cholangiocytes
Ciliated cells
Club cells
Collecting duct cells
Cone photoreceptor cells
Cytotrophoblasts
dendritic cells
Distal enterocytes
Distal tubular cells
Ductal cells
Early spermatids
Endometrial stromal cells
Endothelial cells
Enteroendocrine cells
Erythroid cells
Excitatory neurons
Exocrine glandular cells
Extravillous trophoblasts
Fibroblasts
Gastric mucus−secreting cells
Glandular and luminal cells
granulocytes
Granulosa cells
Hepatocytes
Hofbauer cells
Horizontal cells
Inhibitory neurons
Intestinal goblet cells
Ionocytes
Kupffer cells
Langerhans cells
Late spermatids
Leydig cells
Lymphatic endothelial cells
Macrophages
Melanocytes
Mesothelial cells
Microglial cells
monocytes
Mucus glandular cells
Muller glia cells
NK−cells
Oligodendrocyte precursor cells
Oligodendrocytes
Oocytes
Ovarian stromal cells
Pancreatic endocrine cells
Paneth cells
Peritubular cells
Plasma cells
Prostatic glandular cells
Proximal enterocytes
Proximal tubular cells
Rod photoreceptor cells
Salivary duct cells
Schwann cells
Secretory cells
Serous glandular cells
Sertoli cells
Skeletal myocytes
Smooth muscle cells
Spermatocytes
Spermatogonia
Squamous epithelial cells
Suprabasal keratinocytes
Syncytiotrophoblasts
T−cells
Undifferentiated cells
RAMP1
RAMP2
RAMP3
Expression > 1 nTPM
Yes
No
C
Mesothelial cells
Ovarian stromal cells
Langerhans cells
Melanocytes
Prostatic glandular cells
Ciliated cells
Club cells
Basal respiratory cells
dendritic cells
Inhibitory neurons
Astrocytes
Oligodendrocyte precursor cells
Intestinal goblet cells
Ductal cells
NK−cells
Paneth cells
Oocytes
Proximal enterocytes
Undifferentiated cells
Enteroendocrine cells
Exocrine glandular cells
Cardiomyocytes
Schwann cells
Cholangiocytes
Hepatocytes
Extravillous trophoblasts
monocytes
Syncytiotrophoblasts
Bipolar cells
Distal enterocytes
Proximal tubular cells
Squamous epithelial cells
Rod photoreceptor cells
Cone photoreceptor cells
Cytotrophoblasts
Early spermatids
Late spermatids
Serous glandular cells
Mucus glandular cells
Alveolar cells type 1
Suprabasal keratinocytes
Distal tubular cells
Spermatocytes
Leydig cells
Spermatogonia
Glandular and luminal cells
Peritubular cells
Microglial cells
Muller glia cells
Breast glandular cells
Breast myoepithelial cells
Basal keratinocytes
Plasma cells
Kupffer cells
Salivary duct cells
Secretory cells
Sertoli cells
Hofbauer cells
Gastric mucus−secreting cells
Ionocytes
Basal prostatic cells
granulocytes
Macrophages
Collecting duct cells
Pancreatic endocrine cells
Alveolar cells type 2
B−cells
T−cells
Basal squamous epithelial cells
Granulosa cells
Erythroid cells
Horizontal cells
Excitatory neurons
Oligodendrocytes
Adipocytes
Endothelial cells
Lymphatic endothelial cells
Endometrial stromal cells
Skeletal myocytes
Fibroblasts
Smooth muscle cells
RAMP1
RAMP2
RAMP3
Expression log2(nTPM)
8
6
4
2
0

Fig. S12.

RNA expression of GPCRs and RAMPs in human cells and tissues.

(**A**) Violin plots showing GPCR-RAMP RNA expression ratios in human cells. GPCRs were annotated as RAMP-interacting or not based on the SBA assay results. The expression ratio between each GPCR and each RAMP was calculated across all human cell types with available RNASeq data. Cell types in which either GPCR or RAMP expression was less than 1 nTPM were excluded. The data are plotted as log2 of the GPCR-RAMP expression ratio and the number of datapoints for each dataset is provided at the bottom of each plot. GPCRs without definitive evidence for interaction or non-interaction for each RAMP were excluded; 205 GPCRs out of 215 GPCRs were analyzed in total. Significance was determined by a two-sided unpaired Wilcoxon test (** $p < 0.005$, $p >= 0.05$ if not marked). Sample sizes and p-values are listed in **Table S3**. (**B**) Binary heatmaps are shown for RAMP expression, where each row shows data from a human tissue type (*left*) or single-cell data from a human cell type (*right*). Green, RAMP expression greater than 1 nTPM. Purple, RAMP expression less than 1 nTPM. (**C**) Heatmap is shown based on single-cell data for RAMP expression in different human cell types (*rows*) for each RAMP (*columns*) arranged by hierarchical clustering. Data are from proteinatlas.org (*21, 22*).

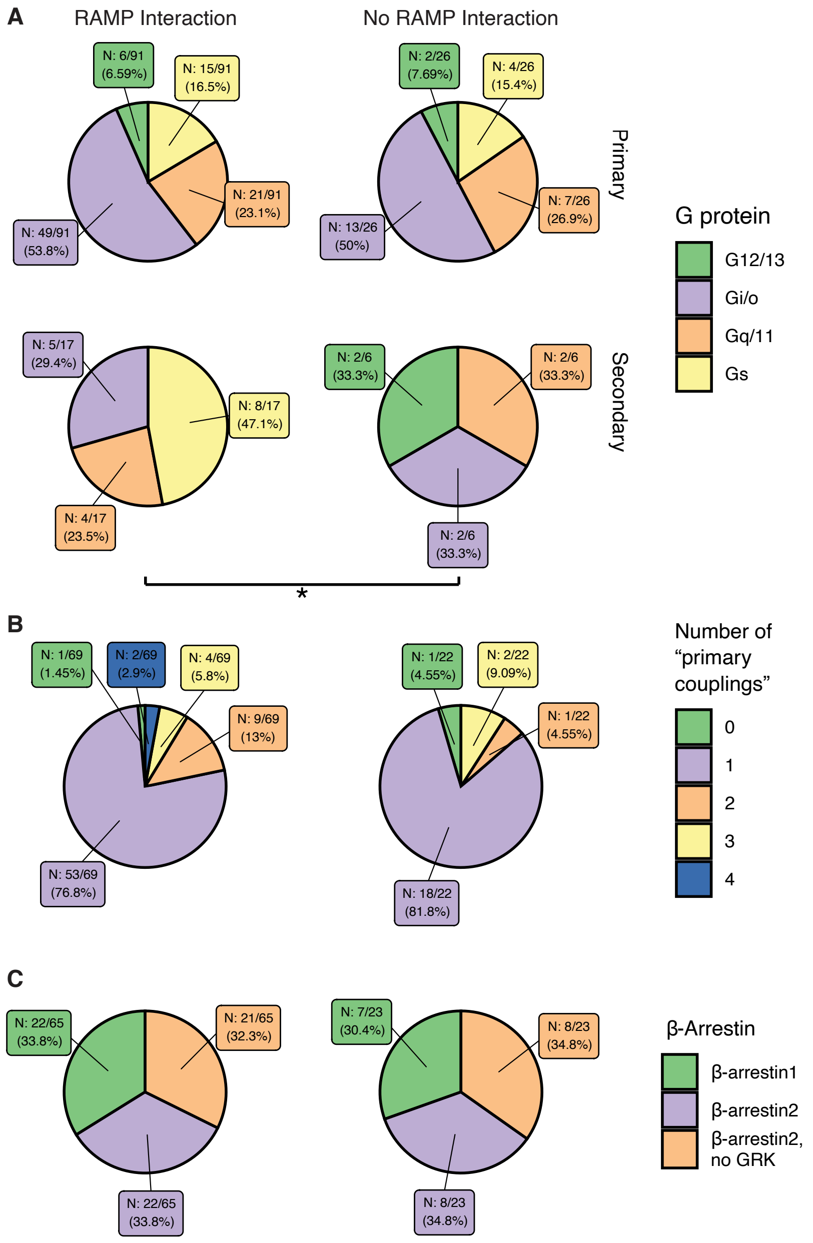

Fig. S13.

G protein and β-arrestin coupling for RAMP-interacting and non-interacting GPCRs.

Pie charts demonstrate the distribution of RAMP interacting and non-interacting GPCRs based on GPCR coupling with downstream effector molecules. (**A**) GPCRs that show evidence for RAMP interaction are classified as "RAMP Interaction" and those that do not are classified as "No RAMP Interaction". G protein subtype is color-coded and G protein coupling for a given GPCR is classified as either primary or secondary. (**B**) The vast majority of GPCRs couple to a single G protein subtype. However, approximately

one in five GPCRs couples to more than one G protein subtype. (**C**) GPCR coupling to β-arrestin subtypes based on published $\log(E_{max}/E_{50})$ values, assigned as no coupling if 0 and coupling if > 0. Significance was determined by the Fisher exact test (* $p < 0.05$, $p >= 0.05$ if not marked, **Table S3**). There were data on G protein coupling for 58 GPCRs and data for both G protein and β-arrestin coupling data for 32 GPCRs (n = 90 in total) out of the 215 GPCRs studied in this paper. All data are from gpcrdb.org (*23-26*).

Table S1.

Antibodies and GPCRs. List of all used antibodies (Ab), their target GPCR or RAMP, and information about each GPCR. Information about which assay each Ab was used in is also specified.

(provided as a separate Excel file)

Table S2.

Fitting parameters of IP1 Dose-response curves. The values provided correspond to the dose-response curves presented in **Fig. S2**, computed in Prism 10. Curves were fit to a log[agonist] versus response three-parameter non-linear model with least squares fit at an alpha of 0.05. Df (degrees of freedom) was computed from the number of samples and groups and indicates the number of independent pieces of information. All other values presented are the calculated best-fit values. EC_{50}, concentration of agonist that provokes a response halfway between the basal and maximum level of stimulation.

	**Basal**	E_{max}	$logEC_{50}$	EC_{50}	**span**	**Df**
**HA-CALCRL-1D4 + RAMP1 (Dose-response CGRP)**						
**FLAG-RAMP1-OLLAS**	4.908	75.32	-8.845	1.429e-9	70.42	21
**3xHA-RAMP1-OLLAS**	6.760	76.02	-8.834	1.467e-9	69.26	21
**HA-CALCRL-1D4 + RAMP1 (Dose-response Adrenomedullin)**						
**FLAG-RAMP1-OLLAS**	5.971	83.69	-7.209	6.186e-8	77.72	21
**3xHA-RAMP1-OLLAS**	6.818	82.96	-6.891	1.286e-7	76.10	21
**HA-CALCRL-1D4 + RAMP2 (Dose-response Adrenomedullin)**						
**FLAG-RAMP2-OLLAS**	4.598	53.48	-8.252	5.592e-9	48.89	21
**3xHA-RAMP2-OLLAS**	4.138	39.29	-8.178	6.631e-9	35.15	21
**HA-CALCRL-1D4 + RAMP3 (Dose-response Adrenomedullin)**						
**FLAG-RAMP3-OLLAS**	4.987	96.09	-8.886	1.300e-9	91.10	21
**3xHA-RAMP3-OLLAS**	4.634	94.19	-9.036	9.194e-10	89.56	21
**FLAG-CALCRL-1D4 + RAMP1 (Dose-response CGRP)**						
**FLAG-RAMP1-OLLAS**	7.213	66.32	-9.254	5.570e-10	59.10	21

**3xHA-RAMP1-OLLAS**	5.488	61.50	-9.304	4.966e-10	56.01	21
**FLAG-CALCRL-1D4 + RAMP1 (Dose-response Adrenomedullin)**						
**FLAG-RAMP1-OLLAS**	7.310	67.88	-7.646	2.257e-8	60.56	21
**3xHA-RAMP1-OLLAS**	6.124	62.39	-7.461	3.456e-8	56.27	21
**FLAG-CALCRL-1D4 + RAMP2 (Dose-response Adrenomedullin)**						
**FLAG-RAMP2-OLLAS**	4.698	91.63	-8.271	5.359e-9	86.93	21
**3xHA-RAMP2-OLLAS**	4.202	79.70	-8.266	5.417e-9	75.70	21
**FLAG-CALCRL-1D4 + RAMP3 (Dose-response Adrenomedullin)**						
**FLAG-RAMP3-OLLAS**	6.015	95.66	-9.051	8.900e-10	89.64	21
**3xHA-RAMP3-OLLAS**	3.705	104.3	-9.075	8.412e-10	100.6	21

Table S3.

Statistical test parameters, sample sizes, and p-values. The figure panel and test type are specified. ANOVA test results are from the aov() and summary() functions. The tables contain the capture method (Capture), degrees of freedom (Df), sums (Sum Sq) and means of squares (Mean Sq), the test statistic (F value), and p-value (Pr(>F)). Dunnett test results are from the DunnettTest() function, testing against samples with mock transfection or empty samples. The tables contain the sample type being tested against (interactor or gpcr_interactor), the capture method (capture), detection method (detection), the number of samples for each sample type being tested (n), the difference in observed means (diff), the 95% confidence interval (lwr.ci, upr.ci) and the p-value (pval). Wilcoxon rank-sum test results are obtained from the wilcox.test() function. The tables contain the comparison being tested (gpcr, ramp), the capture method if applicable (capture), the p-value (p.value) and the test statistic (statistic). All statistical analysis was carried out in R.

(provided as a separate Excel file)

Table S4.
Thresholds for GPCR-RAMP complex detection for each epitope-based capture-detection scheme, per RAMP. Thresholds were determined by the intersection of specificity and sensitivity curves, constructed based on the GPCR-RAMP interactions reported in the literature, and are reported as the R.Z-score. Sensitivity and Specificity values at the selected threshold are reported as a probability between 0 and 1.

Interaction	Capture	Detection	Threshold	Sensitivity	Specificity
**RAMP1**	1D4	OLLAS	-0.60	0.55	0.62
**RAMP2**	1D4	OLLAS	0.18	0.62	0.64
**RAMP3**	1D4	OLLAS	0.92	0.56	0.50
**RAMP1**	FLAG	OLLAS	-0.65	0.64	0.69
**RAMP2**	FLAG	OLLAS	0.11	0.57	0.55
**RAMP3**	FLAG	OLLAS	0.40	0.78	0.75
**RAMP1**	OLLAS	1D4	0.74	0.55	0.62
**RAMP2**	OLLAS	1D4	0.17	0.52	0.55
**RAMP3**	OLLAS	1D4	0.34	0.61	0.75
**RAMP1**	HA	1D4	-0.34	0.36	0.46
**RAMP2**	HA	1D4	1.18	0.48	0.55
**RAMP3**	HA	1D4	0.67	0.56	0.25
**RAMP1**	RAMP1	1D4	0.68	0.36	0.62
**RAMP2**	RAMP2	1D4	3.63	0.43	0.45
**RAMP3**	RAMP3	1D4	3.14	0.56	0.75

Table S5.
GPCR-RAMP complex detection outcomes for previously reported interactions. The number of GPCR-RAMP complexes that are true positives (TP), true negatives (TN), false positives (FP) and false negatives (FN) per capture-detection scheme, per RAMP based on the literature. Anti-1D4, anti-FLAG and anti-GPCR capture (GPCR capture) corresponds to anti-OLLAS detection (RAMP detection). Anti-HA, anti-OLLAS and anti-RAMP capture (RAMP capture) corresponds to anti-1D4 detection (GPCR detection). A dash indicates a threshold was not computed for the capture-detection scheme and RAMP indicated.

Capture		RAMP1	RAMP2	RAMP3
**1D4**	TP	6	13	10
	TN	8	7	2
	FP	5	4	2
	FN	5	8	8
**FLAG**	TP	7	12	14
	TN	9	6	3
	FP	4	5	1
	FN	4	9	4
**HA**	TP	4	10	10
	TN	6	6	1
	FP	7	5	3
	FN	7	11	8
**OLLAS**	TP	6	11	11
	TN	8	6	3
	FP	5	5	1
	FN	5	10	7
**RAMP1**	TP	4	-	-
	TN	8	-	-
	FP	5	-	-
	FN	7	-	-
**RAMP2**	TP	-	9	-
	TN	-	5	-
	FP	-	6	-
	FN	-	12	-
**RAMP3**	TP	-	-	10
	TN	-	-	3
	FP	-	-	1
	FN	-	-	8
**anti-GPCR Abs**	TP	3	13	15
	TN	17	7	0
	FP	2	6	5
	FN	4	5	1

Table S6.

Thresholds for GPCR-RAMP detection for each protein-based capture scheme. The table lists all anti-GPCR Abs used, sorted by GPCR name alphabetically. The threshold and interaction results for each RAMP are provided.

(provided as a separate Excel file)

Table S7.

Summary of GPCR-RAMP complexes detected for each GPCR with each RAMP. The first sheet lists the evidence for interaction (strong, medium, weak) for each GPCR-RAMP pair tested by epitope-based capture (215 GPCRs) and protein-based capture (154 GPCRs). Information on whether a particular interaction is expected based on the literature is also included. Strong evidence corresponds to >66% passing capture-detection schemes. Medium evidence corresponds to 33-66% passing capture-detection schemes. Weak evidence corresponds to <33% passing capture-detection schemes. The second sheet lists the number of passing capture-detection schemes for each GPCR-RAMP pair tested. The third sheet provides the number of passing capture-detection schemes for each GPCR-RAMP pair tested as a fraction out of the total number of capture-detection schemes for the approach specified. There are five capture-detection schemes in total for all epitope-based values. The number of total capture-detection schemes for protein-based capture varies from zero to six.

(provided as a separate Excel file)

Table S8.

Native GPCR-RAMP complexes detected by SBA assay in three human cell lines. Complexes were captured with anti-GPCR antibodies from the Human Protein Atlas and detected with an anti-RAMP antibody specific to each RAMP. Value are Robust Z-scores, calculated separately for each detection method.

**RAMP1 detection**				
**GPCR**	**Antibody**	**Expi**	**SH-SY5Y**	**SK-N-MC**
**ADGRF5**	HPA065251	6.89	25.16	26.59
**CCKAR**	HPA073900	15.60	10.72	7.67
**CCKAR**	HPA051284		4.58	4.61
**CHRM4**	HPA072083	16.32	13.18	4.06
**DRD5**	HPA065783	3.88		
**GLP1R**	HPA065175		4.03	4.57
**GLP2R**	HPA027929		5.83	
**GPR20**	HPA059117	5.73		
**GPR37**	HPA068009		3.55	
**HRH4**	HPA035009	3.54	5.09	
**PTGDR**	HPA049668	3.91	4.99	3.71
**VIPR2**	HPA062707	4.25		

**RAMP2 detection**				
**GPCR**	**Antibody**	**Expi**	**SH-SY5Y**	**SK-N-MC**
**ADGRF5**	HPA065251		10.60	6.41
**CCKAR**	HPA073900	14.74	8.99	6.08

**CHRM4**	HPA072083	10.80	8.92	
**CRHR2**	HPA073345	13.50		
**DRD5**	HPA065783	7.68	5.74	5.74
**DRD5**	HPA013797	12.20		7.34
**GCGR**	HPA077370			3.54
**GLP1R**	HPA065175		4.14	3.97
**GLP2R**	HPA027929		4.50	
**GPR37**	HPA068009	3.84		
**HRH4**	HPA035009		4.59	
**PTGDR**	HPA049668	3.81	5.75	
**SCTR**	HPA007312	4.92		
**VIPR2**	HPA062707	4.57		

**RAMP3 detection**				
**GPCR**	**Antibody**	**Expi**	**SH-SY5Y**	**SK-N-MC**
**ADGRF5**	HPA065251	4.04	8.72	6.39
**ADRA2C**	HPA057688			3.91
**ADRB3**	HPA065408		4.80	9.56
**CCKAR**	HPA073900	11.69	6.94	4.83
**CCKAR**	HPA051284		3.79	
**CHRM4**	HPA072083	9.11	7.23	
**CXCR2**	HPA031999			4.02
**DRD5**	HPA065783	3.64		
**GLP1R**	HPA065175		4.09	4.20
**GLP2R**	HPA027929		5.45	
**GPR37**	HPA068009	3.58		
**GPR4**	HPA019207			8.56
**GPR61**	HPA026088	4.06		7.27
**GRM2**	HPA027868	4.00	4.59	4.14
**HCAR2;HCAR3**	HPA028660		3.99	
**HRH4**	HPA035009		4.24	
**HTR1F**	HPA005555		4.24	5.85
**MCHR2**	HPA030115	9.89	5.91	
**OPN3**	HPA049243		3.54	6.31
**OPRM1**	HPA067435		3.74	5.38
**PTGDR**	HPA049668	3.72	4.80	
**PTH2R**	HPA010534			6.28
**S1PR1**	HPA048588			3.85
**SCTR**	HPA007312	3.96		
**SSTR4**	HPA064252	3.98	4.07	
**VIPR2**	HPA062707	4.34		
